# Cost-effectiveness of providing pre-exposure and post-exposure prophylaxis for the prevention of HIV via online pharmacies in western Kenya: a modelling study

**DOI:** 10.1016/j.langlo.2026.103977

**Published:** 2026-08-07

**Authors:** Akash Malhotra, Nishali Patel, David Kaftan, Yilin Chen, Cory Arrouzet, Arden Saravis, Catherine Kiptinness, Tabitha Kareithi, Kenneth Ngure, Katrina F Ortblad, Monisha Sharma

**Affiliations:** a Department of Global Health, University of Washington, Seattle, WA, USA; b Institute for Health Metrics and Evaluation, University of Washington, Seattle, WA, USA; c Department of Population Health, New York University Grossman School of Medicine, New York, NY, USA; d CHOICE Institute, School of Pharmacy, University of Washington, Seattle, WA, USA; e Department of Epidemiology, University of Washington, Seattle, WA, USA; f Partners in Health and Research Development, Thika, Kenya; g Public Health Science Division, Fred Hutchinson Cancer Center, Seattle, WA, USA

## Abstract

**Background:**

Oral pre-exposure prophylaxis (PrEP) and post-exposure prophylaxis (PEP) provision via online pharmacies (henceforth online PrEP and PEP) offers promise in increasing biomedical HIV prevention coverage. In this study, we aimed to estimate the cost-effectiveness of online PrEP and PEP scale-up in western Kenya.

**Methods:**

We adapted a network-based model, EMOD-HIV, to simulate online PrEP and PEP implementation from 2026 to 2036; costs and use of online PrEP and PEP and client characteristics were informed by the ePrEP Kenya pilot study, which evaluated online PrEP and PEP provision in Nairobi and Mombasa, Kenya. Other parameters were obtained from surveillance data and published literature. Eligible clients were aged 15–49 years reporting condomless sex with non-marital partners. We assumed online PrEP and PEP provision was implemented through public–private partnership, with the Kenya Ministry of Health providing PrEP and PEP drugs and service delivery costs paid by the online pharmacy (and charged to clients). We estimated HIV infections, HIV-related deaths, and disability-adjusted life-years (DALYs) averted compared with the baseline scenario of background oral PrEP only. We calculated incremental cost-effectiveness ratios (ICERs) assessing only costs incurred by the Kenya Ministry of Health over 35 years and used a supply-side threshold of US$500 per DALY averted. We calculated 95% uncertainty intervals (UIs) across 100 parameter sets.

**Findings:**

Online PrEP and PEP was projected to reach population coverage of 0·7% (95% UI 0·7–0·8) for PEP and 0·3% (0·2–0·3) for PrEP and avert 13·9% (10·1–17·1) of HIV infections over 10 years. HIV-related deaths were reduced by 4·3% (95% UI 2·0–6·9) over the 35-year time horizon. The intervention was cost-effective from the Kenya Ministry of Health perspective (ICER $212 [95% UI 15–1409] per DALY averted). In a scenario assuming only online PEP availability (to isolate the effect of PEP), 11·6% (95% UI 8·6–15·2) of HIV infections were averted (ICER $210 [95% UI 41–3798] per DALY averted). The intervention remained cost-effective when varying PrEP and PEP effectiveness and assuming HIV testing and treatment disruptions.

**Interpretation:**

Online PrEP and PEP can avert substantial HIV infections, even with low population coverage. PEP was responsible for most intervention health benefits, likely due to high observed demand for PEP compared with PrEP in the pilot study. Leveraging private retailers can be efficient for scaling up HIV prevention in an era of shrinking donor funding.

**Funding:**

Gates Foundation.

## Introduction

Both oral pre-exposure prophylaxis (PrEP) and post-exposure prophylaxis (PEP) are promising HIV prevention strategies that can be scaled up to reduce HIV incidence in eastern and southern Africa. Daily oral PrEP is more than 90% effective when used with high adherence and PEP is 90–95% effective if initiated 48 h or sooner after potential HIV exposure.^1^ Since 2015, oral PrEP has been recommended by WHO for people at substantial HIV risk and many countries in eastern and southern Africa have rolled out PrEP in clinics.^2^ However, clinic-based PrEP uptake and continuation is low in eastern and southern Africa, with clients citing barriers including travel distance, waiting times, stigma, privacy concerns, and inconvenient operating hours.^3^ Similarly, PEP availability is generally limited to clinics and uptake is low. WHO recommends differentiated, community-centred delivery of HIV biomedical prevention with task shifting to increase accessibility.^4^ Studies show PrEP provision in community-based settings, including pharmacies, achieves high uptake and reaches individuals not accessing clinic-based services.^5^^,^^6^ Modelling studies have found community-based PrEP and PEP are cost-effective in eastern and southern Africa.^7^, ^8^, ^9^

**Research in context**

**Evidence before this study**

We searched PubMed for articles published from Jan 1, 2016, to Sept 15, 2025, assessing the health or economic effect of pre-exposure prophylaxis (PrEP) and post-exposure prophylaxis (PEP) in sub-Saharan Africa using the terms: ((“HIV”[tiab]) AND “Africa” AND (“pre-exposure prophylaxis”[tiab] OR “PrEP”[tiab] OR “post-exposure prophylaxis”[tiab] OR “PEP”[tiab]) AND (“economic evaluation”[tiab] OR “cost∗”[tiab] OR “budget impact”[tiab])). Our search identified 181 articles, including 23 studies that modelled the cost-effectiveness of PrEP in Africa, and two studies that modelled PEP in Africa. Among the PEP studies, one study, by Phillips and colleagues, explored the cost-effectiveness of broad community availability of tenofovir, lamivudine, and dolutegravir across Africa, assuming self-selection among those at HIV risk, and the other, by Garnett and colleagues, conducted a back-of-the-envelope analysis of PEP cost-effectiveness in eastern and southern Africa by use of theoretical assumptions. We did not find any studies modelling the cost-effectiveness of PEP by use of utilisation data from real-world studies.

**Added value of this study**

To our knowledge, this is the first study to evaluate the health and economic effect of PEP provision in Africa using empirical data on client use and the first to evaluate the cost-effectiveness of PrEP and PEP provision via an online pharmacy. We find that online PrEP and PEP provision can avert substantial HIV incidence with low population coverage and is cost-effective from the Kenya Ministry of Health perspective. Online PEP accounts for most of the intervention health benefits, likely due to the high observed PEP demand in the pilot study.

**Implications of all the available evidence**

Online PrEP and PEP provision has the potential to efficiently increase coverage of biomedical HIV prevention in western Kenya and similar settings. PEP is an underused prevention tool at high demand that can complement PrEP scale-up. Policy makers could consider integrating PEP and PrEP in differentiated delivery models and consider public–private partnerships to maximise reach of HIV prevention in the context of constrained budgets and donor transition.

Leveraging online pharmacies for oral PrEP and PEP provision (henceforth online PrEP and PEP) is a novel strategy that can expand HIV prevention coverage by providing convenient and discreet community-based services. Online pharmacies offer direct-to-consumer delivery of medications, health products, health information, and telehealth consultations. Telehealth services have grown rapidly in eastern and southern Africa, with increasing internet availability and mobile phone ownership; growth was expedited by the COVID-19 pandemic, during which HIV services were successfully delivered remotely.^10^ The ePrEP Kenya pilot study evaluated the first strategy for online pharmacy-based PrEP and PEP delivery in eastern and southern Africa.^11^ The strategy involved clients having remote consultations with clinical providers to ensure PrEP or PEP eligibility, ordering courier-delivered HIV self-testing kits or provider-administered HIV self-testing kits to verify HIV-negative status, receiving HIV drugs delivered at home or a convenient location, and having access to virtual user support. The pilot found online pharmacy-based PrEP and PEP was feasible and acceptable to clients and providers and reached PrEP-naive populations at HIV risk.^12^^,^^13^ Additionally, clients were willing to pay for online PrEP and PEP services.^14^ Online PEP was particularly in demand, with five-times as many clients accessing PEP compared with PrEP, and clients choosing repeat PEP over transitioning to PrEP.^12^ The observed preference for PEP over PrEP could be due to its event-driven nature; PEP is easier to align with potential HIV exposures compared with PrEP, which necessitates anticipation of future condomless sex. Given the importance of timely PEP initiation, online pharmacies can be particularly well suited to delivering PEP within the effectiveness window due to longer operating hours (including evenings and weekends); in the ePrEP Kenya pilot study, 629 (96%) of 653 clients received drugs within 72 h of potential exposure.^12^ Online pharmacies can also be leveraged to provide long-acting products in the pipeline including monthly oral PrEP.

Estimating the health and economic effects of online PrEP and PEP provision is essential to inform policy decisions regarding scale-up. Online PrEP and PEP service delivery will likely require partnerships between private and government sector agencies since PrEP and PEP drugs are often provided by ministries of health, whereas service delivery costs are generally paid by the pharmacy (and charged to clients). Private–public partnerships to distribute government commodities (ie, PrEP and PEP drugs) are likely efficient strategies, particularly in an era of reduced donor funding for HIV. In this analysis, we aimed to estimate cost-effectiveness of online PrEP and PEP from the Kenya Ministry of Health perspective using costs and online PEP and PrEP uptake by demographics and sexual behaviour from the ePrEP Kenya pilot. To our knowledge, this is the first study to estimate online PrEP and PEP cost-effectiveness and the first to assess PEP cost-effectiveness by use of primary data on client characteristics.

## Methods

### Data sources

We used costs and online PrEP and PEP uptake data from the ePrEP Kenya pilot study conducted in Nairobi and Mombasa counties.^12^^,^^15^ Briefly, this single-arm pilot was conducted from Oct 24, 2022, to Dec 31, 2023, in collaboration with MYDAWA, Kenya’s first online pharmacy. Eligible clients were aged 18 years or older, reported behaviours associated with HIV risk or a recent HIV exposure, and were willing to pay for HIV testing (∼US$2 for HIV self-testing or ∼$1 for provider-administered HIV testing) and courier delivery of HIV testing kits and PrEP or PEP drugs (∼US$1 per delivery).^14^ Online PrEP and PEP was advertised on MYDAWA’s website on the sexual wellness page; interested individuals clicked the advertisement and self-screened for HIV risk. Eligible clients attended telehealth visits with online pharmacy health-care providers and received counselling and screening for PrEP or PEP. Clients were prescribed PEP if they reported potential HIV exposure within the past 72 h, or PrEP if reporting ongoing HIV risk. Clients had HIV testing kits delivered by courier to their setting of choice to verify HIV-negative status and received courier-delivered PEP (28-day supply) or PrEP (30-day supply for first initiation of PrEP, and 90-day supply thereafter).

Overall, 1549 (88·2%) of 1757 enrolled individuals received online PEP and 208 (11·8%) received PrEP. 1121 (63·8%) clients were male, 636 (32·2%) were female, and 1273 (72·5%) were age 25 years or older (table 1).^12^ All demographic and sexual behaviour variables were self-reported via surveys. We used PrEP continuation data from all individuals who continued PrEP, regardless of whether they originally initiated PrEP or PEP. Data used for this modelling study can be found in appendix 1 (pp 3–30).

**Table 1:** Characteristics of clients in the ePrEP Kenya pilot study

	Clients who received online PrEP (n=208)	Clients who received online PEP (n=1549)
**Demographics**		
Median age, years	28 (25–34)	27 (24–33)
Age 25 years or older	154 (74·0%)	1119 (72·2%)
Sex		
Male	155 (74·5%)	966 (62·4%)
Female	53 (25·5%)	583 (37·6%)
Married	24 (11·5%)	196 (12·7%)
**Behaviours associated with risk of HIV acquisition in the past 6 months**		
Multiple concurrent sexual partners	136 (65·4%)	716 (46·2%)
One or more partners of unknown HIV status	153 (73·6%)	1384 (89·3%)
One or more partners living with HIV	24 (11·5%)	42 (2·7%)
Transactional sex	6 (2·9%)	41 (2·6%)

Data are n (%) or median (IQR). PEP=post-exposure prophylaxis. PrEP=pre-exposure prophylaxis.

### Model design and parameters

We adapted a network-based model, EMOD-HIV,^16^ which incorporates population demographics, HIV natural history, and heterosexual HIV transmission, configured to match age-specific and sex-specific sexual relationship patterns. The model includes an HIV care cascade with HIV testing, linkage and retention in care, and a PrEP cascade with uptake, adherence, persistence, and reinitiation.^17^ We modified the model to incorporate a PEP cascade and assumed individuals can seek PEP after condomless sex with non-marital partners.^7^ The model was parameterised with empirical data from western Kenya (Siaya, Kisumu, Homa Bay, Migori, Kisii, and Nyamira counties), including fertility, mortality, voluntary male circumcision coverage, and number of people on oral PrEP (appendix 1 pp 12–30) and calibrated to data on HIV prevalence and antiretroviral therapy (ART) coverage from western Kenya. We conducted the modelling using 100 good-fitting model parameter sets over monthly time steps.

In the baseline (ie, no online PrEP and PEP) scenario, we assumed background daily oral PrEP use at currently observed coverage for Kenya among eligible individuals (defined as those aged 15–49 years with HIV risk indication [ie, individuals reporting condomless sex with non-marital partners]).^18^ We assumed oral PrEP decreased HIV acquisition risk by 75%, accounting for average adherence, and 3-month PrEP persistence based on real-world studies (table 2).^19^ In intervention scenarios, online PrEP and PEP was implemented from 2026 to 2036 to evaluate the health and economic effects of a 10-year delivery; we used a 35-year time horizon (2026–60) to estimate long-term outcomes and cost-effectiveness.

**Table 2:** Model parameters, assumptions, and cost inputs

	Value	Source
**Model assumptions**		
Oral PrEP effectiveness	75%	Baeten et al (2012)^19^
PEP effectiveness	95%	Zhang et al (2025)^1^
Online pharmacy PrEP scale-up period	2026–29	Assumption
Online PrEP and PEP implementation period	2026–35	Assumption
Analytical time horizon	2026–60	Assumption
**Costs:KenyaMinistry of Health perspective**		
PrEP drugs (30-day supply)	$4·92	Chen et al (2025);^20^ includes 15% supply chain costs
PEP drugs (28-day supply)	$6·94	Chen et al (2025);^20^ includes 15% supply chain costs
Long-acting monthly oral PrEP (cost per pill)	$2·88	Assumed at $2·50 per pill plus 15% supply chain costs (based on expert opinion)
Background facility-based oral PrEP (monthly)	$10·88	Wanga et al (2021)^21^
Annual health-care costs among people living with HIV who are not on ART		
CD4 count <200 cells per μL	$110·30	Eaton et al (2014)^22^
CD4 count 200–349 cells per μL	$30·38	Eaton et al (2014)^22^
CD4 count >350 cells per μL	$8·59	Eaton et al (2014)^22^
End-of-life care per person	$105·68	Eaton et al (2014)^22^
Annual ART provision per person∗	$196·85	Larson et al (2018),^23^ Long et al (2010),^24^ The Global Fund to Fight AIDS, Tuberculosis and Malaria (2024),^25^ and Haas et al (2015)^26^
Facility-based testing: cost per HIV-positive test	$3·68	Meisner et al (2021)^27^
Facility-based testing: cost per HIV-negative test	$2·64	Meisner et al (2021)^27^
**Costs: online pharmacy perspective**		
HIV self-testing (cost per delivery)	$4·05	Chen et al (2025)^20^
**Costs: client perspective**		
HIV-self testing delivery fee (cost per delivery)	$1·00	Chen et al (2025)^20^
PrEP and PEP drug delivery fee (cost per delivery)	$1·00	Chen et al (2025)^20^

Costs are in US$ and are adjusted for inflation and gross domestic product per capita ratio where applicable (appendix 1 pp 7–10). Additional details on costing methods are available in appendix 1 (pp 7–10). ART=antiretroviral therapy. PEP=post-exposure prophylaxis. PrEP=pre-exposure prophylaxis.

∗ ART costs include delivery costs and assume 3% of patients receive second-line ART; different sources provided the various cost components.

For the main scenario and sensitivity analyses, we calibrated the model to fit primary data from online PrEP and PEP clients separately by age, sex, and sexual behaviour in each subgroup (multiple partners, concurrent partners, and serodiscordant relationships; appendix 1 p 4). We parameterised the model with PrEP continuation, PEP-to-PrEP transition, and relative uptake of PEP versus PrEP from the ePrEP Kenya pilot study.

For the online PrEP cascade, eligible individuals first completed HIV self-testing to verify negative status; those testing HIV-positive were assumed to link to ART at background rates. People testing HIV-negative received a 30-day supply of PrEP (tenofovir disoproxil fumarate plus emtricitabine) and had 30% probability of returning for 90-day supply PrEP refills thereafter (resulting in mean PrEP duration of 2·2 months, consistent with the pilot). Individuals completed HIV self-testing at each refill visit. PrEP discontinuation occurred due to loss to follow-up or if eligibility criteria were no longer met. We assumed online PrEP had the same adherence-adjusted PrEP effectiveness as background PrEP (75%).

For the online PEP cascade, people had a probability (as defined in appendix 1 p 3) of initiating PEP after condomless sex with a non-marital partner (based on their age-specific and sex-specific behaviour). We assumed 95% PEP effectiveness based on a modelling study which used pharmacokinetic and viral dynamics, that estimated that a three-drug PEP regimen (tenofovir, lamivudine, and dolutegravir) was more than 95% effective if taken daily for 28 days or more within 48 h of exposure.^1^ Based on the ePrEP Kenya pilot study, we assumed 6% of PrEP-eligible PEP users transitioned to PrEP.^12^ Individuals who discontinued PrEP or PEP could re-initiate at the same probability based on their age-specific and sex-specific behaviour (as defined in appendix 1 p 3).

### Sensitivity analyses

To isolate the health and economic effects of PEP, we ran a scenario of only online PEP provision (without online PrEP). We also lowered PEP efficacy to 75% to assess the effect of reduced adherence or initiation 72 h or less after exposure (instead of less than 48 h, as in the main scenario). We also ran an optimistic scenario assuming 95% PrEP effectiveness to reflect high adherence. To understand the effects of anticipated novel long-acting PrEP and PEP products, we evaluated scenarios replacing daily oral PrEP and PEP with a hypothetical monthly PrEP/PEP, based on the investigational drug MK-8527 (an oral pill taken once per month that is in phase 3 clinical trials; NCT07044297 and NCT07071623), for both online PrEP and PEP; we assumed this hypothetical long-acting monthly PrEP/PEP to have 95% efficacy based on the efficacy of oral PrEP when used at high adherence. We also assessed the effects of potential future President’s Emergency Plan for AIDS Relief (PEPFAR) funding disruptions: we evaluated one scenario in which HIV testing was reduced by 41% and a second in which both HIV testing and ART coverage were reduced by 41%, based on assumptions from a 2025 modelling analysis (as PEPFAR accounts for 41% of Kenya’s HIV budget);^28^ we evaluated a third scenario in which the Kenya Ministry of Health covers 50% of funding gaps from PEPFAR interruptions, resulting in 20·5% reduction in HIV testing and ART. Finally, to assess the effects of our assumptions regarding uptake, we conducted sensitivity analyses assuming all individuals with non-marital partners were eligible for PrEP and PEP and had the same initiation probability. We varied PEP coverage from low to high (ranging from 2% to 5%; appendix 1 p 31) to assess the relationship between targeting and coverage on health benefits (ie, HIV infections, HIV-related deaths, and disability-adjusted life-years [DALYs] averted) and cost-effectiveness.

### Model outcomes

For intervention scenarios, we estimated HIV infections, HIV-related deaths, and DALYs averted with online PrEP and PEP compared with baseline scenario of background oral PrEP only. HIV infections averted are reported for people aged 15–65 years over 10 years of online PrEP and PEP implementation; HIV-related deaths and DALYs averted were calculated over the 35-year time horizon. PrEP and PEP coverage were calculated as the number of person-months on PrEP or PEP divided by the population (eligible individuals aged 15–49 years) person-months during the analysis period and reported for people aged 15–49 years over 10 years of implementation. We also assessed repeat PEP use by client characteristics. We calculated 95% uncertainty intervals (UIs) across 100 parameter sets to assess uncertainty. Model outputs were analysed in R version 4.5.1.

#### Cost-effectiveness and budget impact analysis

We conducted the economic evaluation from the Kenya Ministry of Health perspective, assuming online PrEP and PEP provision was implemented via public–private partnership, in which the Kenya Ministry of Health provides PrEP and PEP drugs while the online pharmacy charges clients to cover HIV testing and service delivery costs. This approach is consistent with the current online PrEP and PEP delivery model via MYDAWA in which clients are charged $6·18 per visit for services, covering clinical consultation and delivery of medicines and test kits; PrEP and PEP drugs are free of charge. Client volume remained stable after the end of the ePrEP Kenya pilot study and implementation of the payment model (appendix 1 pp 11–12; Saidi S, MYDAWA, personal communication). In addition to PrEP and PEP drugs, we assumed the Kenya Ministry of Health incurred costs of background HIV testing, ART, and HIV-related hospitalisations. In the sensitivity analysis, due to uncertainty in future MK-8527 costs, we varied per-pill costs ($2·88, $5·76, and $11·52), accounting for pill price plus 15% supply chain mark-up. Since the price of MK drugs has not been established, we assumed a wide range to encompass likely prices. These numbers are consistent with expert opinion. For cost-effectiveness analyses, we discounted costs and health outcomes by 3% annually, consistent with established WHO guidelines,^29^ and used a commonly referenced supply-side threshold of $500 per DALY averted.^30^ We also estimated the undiscounted 5-year and 10-year budget impact of online PrEP and PEP from the Kenya Ministry of Health perspective. We calculated incremental cost-effectiveness ratios (ICERs), as the difference in cost divided by the difference in effectiveness of the intervention compared with the baseline over a 35-year time horizon.

### Role of the funding source

The funders of the study had no role in study design, data collection, data analysis, data interpretation, or writing of the report.

## Results

Modelled intervention scale-up to the eligible target population resulted in population coverage of 0·7% (95% UI 0·7–0·8) for online PEP and 0·3% (0·2–0·3) for online PrEP (table 3). In the main scenario, online PrEP and PEP provision was projected to reduce HIV infections by 13·9% (95% UI 10·1–17·1) over 10 years of implementation compared with the baseline scenario. HIV-related deaths were reduced by 4·3% (95% UI 2·0–6·9) over the 35-year time horizon. The intervention resulted in 4·78 million (95% UI 4·56–5·10) doses of PEP and 1·72 million (95% UI 1·66–1·80) doses of PrEP dispensed over 10 years. Across subgroups, most modelled online PEP clients used PEP once (51–82%), whereas a smaller proportion initiated twice (16–27%). Repeat PEP use was most common in women aged 25–49 years (appendix 1 p 34). From the Kenya Ministry of Health perspective (ie, only including intervention costs of PrEP or PEP drugs and supply chain costs), the main scenario had an ICER of $212 (95% UI 15–1409) per DALY averted. When isolating the effect of PEP (ie, scenario with online PEP availability only and no online PrEP), coverage of biomedical prevention was reduced by 30% compared with the main scenario (ie, 0·7% PEP and 0·3% PrEP in the main scenario *vs* 0·7% in the PEP only scenario) but health benefits were reduced by only 11·6–16·5% (eg, HIV infections averted was 13·9% in the main scenario *vs* 11·6% in the PEP only scenario, and HIV-related deaths averted were 4·3% in the main scenario *vs* 3·8% in the PEP only scenario); the ICER was similar to the main scenario ($210 [95% UI 41–3798] per DALY averted).

**Table 3:** Health and economic impact of online PrEP and PEP in western Kenya

	Main scenario	Only online PEP available (ie, no online PrEP)	Higher PrEP effectiveness (ie, 95% effectiveness)	Lower PEP effectiveness (ie, 75% effectiveness)	PEPFAR reduction: HIV testing reduced by 41%	PEPFAR reduction: HIV testing and ART coverage reduced by 41%	Main scenario with full payer perspective (theoretical)
PEP coverage	0·7% (0·7 to 0·8)	0·7% (0·7 to 0·8)	0·7% (0·7 to 0·8)	0·7% (0·7 to 0·8)	0·7% (0·7 to 0·8)	0·7% (0·6–0·7)	0·7% (0·7 to 0·8)
PrEP coverage	0·3% (0·2 to 0·3)	··	0·3% (0·2 to 0·3)	0·3% (0·2 to 0·3)	0·3% (0·2 to 0·3)	0·2% (0·2–0·3)	0·3% (0·2 to 0·3)
PEP doses, millions	4·78 (4·56 to 5·10)	4·88 (4·68 to 5·22)	4·78 (4·55 to 5·10)	4·77 (4·55 to 5·11)	4·78 (4·55 to 5·11)	4·56 (4·35–4·87)	4·78 (4·56 to 5·10)
PrEP doses, millions	1·72 (1·66 to 1·80)	0·27 (0·26 to 0·29)	1·73 (1·66 to 1·79)	1·72 (1·65 to 1·79)	1·72 (1·65 to 1·79)	1·65 (1·57–1·71)	1·72 (1·66 to 1·80)
HIV infections averted	13·9% (10·1 to 17·1)	11·6% (8·6 to 15·2)	14·2% (11·0 to 17·9)	12·5% (9·0 to 16·2)	13·7% (10·3 to 17·3)	11·8% (9·0–14·0)	13·9% (10·1 to 17·1)
HIV-related deaths averted	4·3% (2·0 to 6·9)	3·8% (1·5 to 6·0)	4·4% (1·7 to 6·3)	4·2% (1·3 to 6·2)	4·3% (2·3 to 7·1)	4·6% (3·3–5·8)	4·3% (2·0 to 6·9)
DALYs averted	36 304 (–39 887 to 108 288)	22 686 (–27 412 to 109 318)	35 798 (–35239 to 108 398)	32 178 (–24 871 to 103 770)	41 246 (–21 092 to 110 857)	194 502 (97 599–260 461)	36 304 (–39887 to 108 288)
ICER	$212 (15 to 1409)	$210 (41 to 3798)	$178 (22 to 1831)	$260 (31 to 3865)	$198 (20 to 1671)	Cost-saving	$771 (250 to 4798)
5-year budget impact, millions	$16·85 (15·71 to 17·83)	$14·27 (13·21 to 15·37)	$16·78 (15·75 to 17·76)	$16·83 (15·81 to 17·92)	$16·66 (15·73 to 18·01)	$16·14 (14·86–17·15)	$26·69 (25·19 to 28·20)

Costs are in US$. Values in parentheses show 95% uncertainty intervals representing the 2·5th and 97·5th percentiles across 100 parameter sets. Health benefits (ie, HIV infections, HIV-related deaths, and DALYs averted) are compared to the baseline scenario of daily oral PrEP only. HIV infections and DALYs averted are reported for people aged 15–65 years over 10 years of online PrEP and PEP implementation; HIV-related deaths averted are calculated over the 35-year time horizon. PEP and PrEP coverage are reported for people aged 15–49 years over 10 years of implementation. ART=antiretroviral therapy. DALYs=disability-adjusted life-years. ICER=incremental cost-effectiveness ratio. PEP=post-exposure prophylaxis. PEPFAR=President’s Emergency Plan for AIDS Relief. PrEP=pre-exposure prophylaxis.

Most sensitivity analyses yielded findings consistent with the main scenario. Assuming higher PrEP adherence-based effectiveness (95% *vs* 75% in the main scenario), resulted in slightly higher health benefits (14·2% [95% UI 11·0–17·9] of HIV infections averted) and a lower ICER ($178 [22–1831] per DALY averted). Conversely, assuming lower PEP effectiveness (75% *vs* 95% in the main scenario), slightly reduced health benefits (12·5% [95% UI 9·0–16·2] of HIV infections averted) and increased the ICER to $260 (31–3865) per DALY averted. In PEPFAR funding reduction scenarios, the intervention had greater health benefits and more favourable ICERs, particularly in the scenario assuming 41% reduction in both HIV testing and ART, in which online PrEP and PEP averted more than five-times more DALYs than the main scenario and was cost-saving (table 3). The intervention remained cost-saving assuming the Kenya Ministry of Health would cover 50% of funding gaps (appendix 1 p 31). Evaluating a theoretical full payer scenario that includes all costs (incurred by the Kenya Ministry of Health, online pharmacy, and clients) resulted in a higher ICER of $771 (95% UI 250–4798) per DALY averted. Assuming online pharmacies replaced daily oral PrEP and PEP with long-acting monthly oral PrEP resulted in similar health effects as the scenario with PrEP at 95% effectiveness and was cost-saving at long-acting monthly oral PrEP costs of $2·88 per pill. Long-acting monthly oral PrEP remained cost-effective at per-pill costs of $5·76 (ICER $269 [14–2356] per DALY averted) but exceeded the cost-effectiveness threshold at higher costs of $11·52 per pill (ICER $1231 [324–11 159] per DALY averted; appendix 1 p 32). Assuming uniform uptake of online PrEP and PEP among all individuals with a non-marital partner resulted in lower health benefits and higher ICERs than the main scenario; for example, at online PrEP coverage of 0·3% and PEP coverage of 0·6%, the intervention averted 8·8% (95% UI 4·2–12·5) of HIV infections and had an ICER of $440 (165–6703) per DALY averted. Higher coverages of uniform targeting increased health benefits but had higher ICERs (appendix 1 p 32). Additional results (number of HIV infections and deaths averted, 35-year incremental costs, etc) are presented in appendix 1 (pp 33–37).

**Figure 1:**
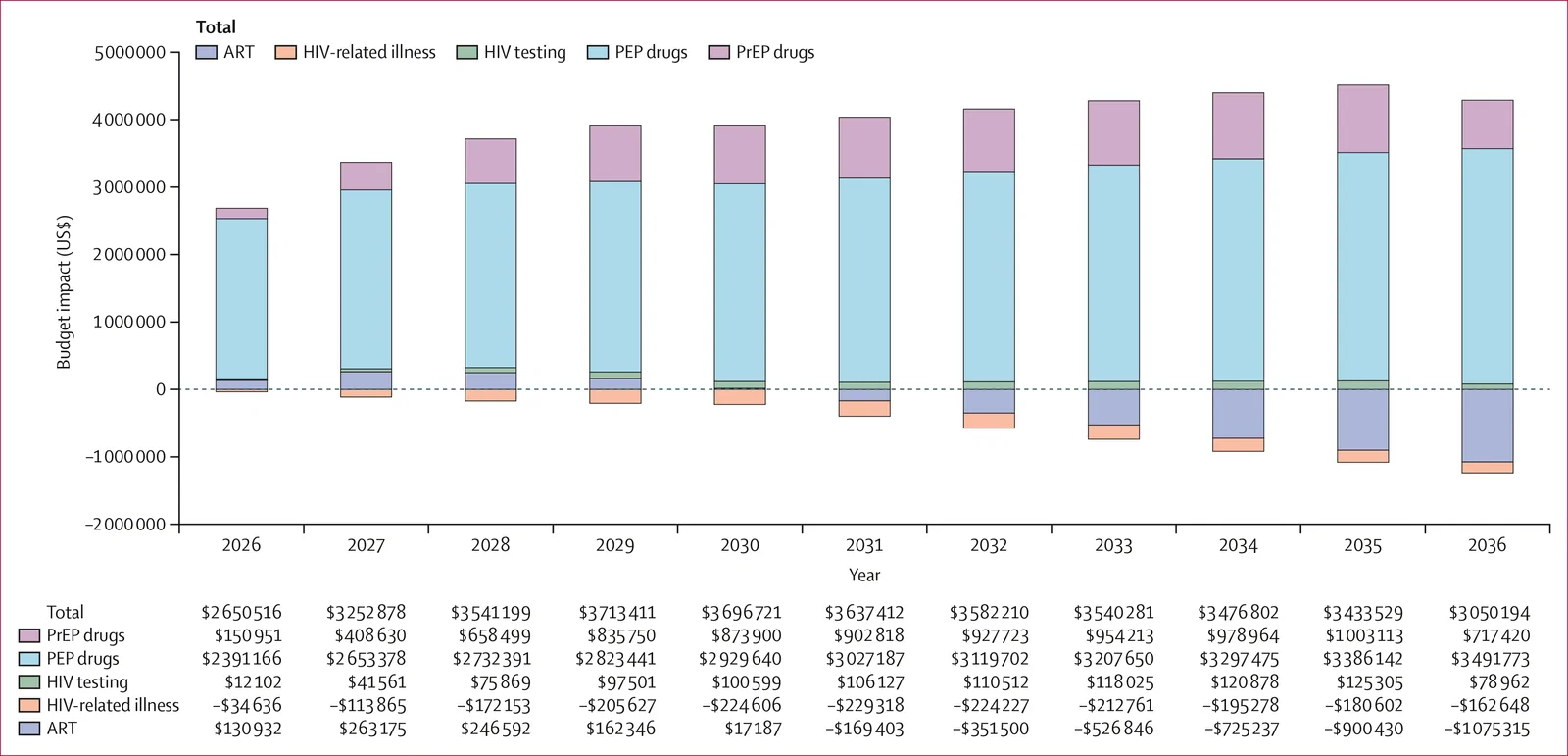
Budget impact analysis Undiscounted 10-year budget impact of implementing online PrEP and PEP from the Kenya Ministry of Health perspective. Costs are in US$. Negative values indicate cost savings. ART=antiretroviral therapy. PrEP=pre-exposure prophylaxis. PEP=post-exposure prophylaxis.

**Figure 2:**
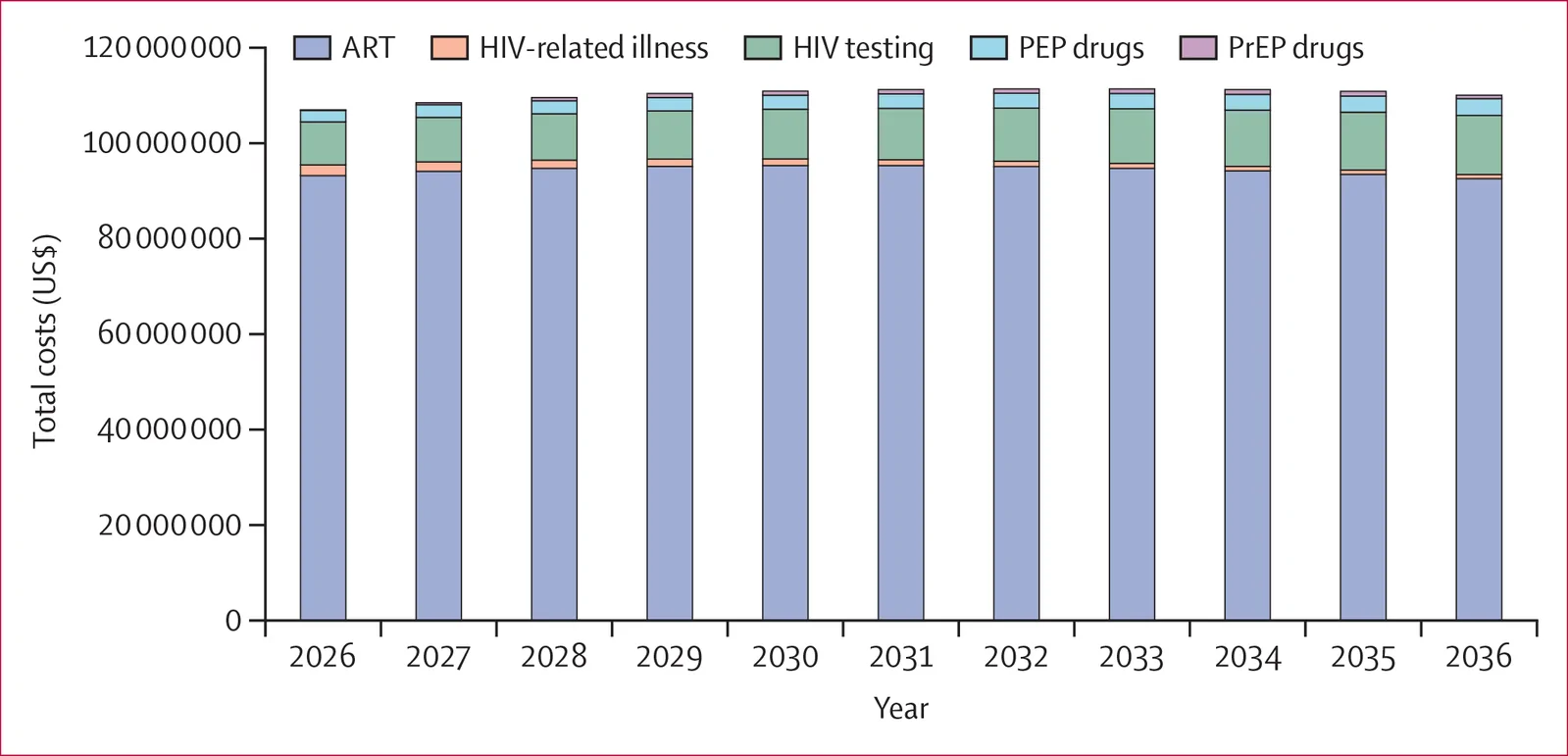
Total costs ART=antiretroviral therapy. PrEP=pre-exposure prophylaxis. PEP=post-exposure prophylaxis.

From the Kenya Ministry of Health perspective, the annual budget impact of implementing online PrEP and PEP ranged from US$2·7–3·8 million, assuming a modelled population of 6·3 million adults (figure 1). Costs increased in the first 3 years as the intervention was scaled up (ie, 2026–28); PEP drugs made up most costs (77%) with PrEP drugs accounting for 20% over this time period. In initial years (ie, 2026–30), the intervention increased ART costs as people with HIV were identified through HIV testing for PrEP or PEP initiation and linked to care; however, in later years (2031 onwards) cost savings in ART and HIV-related illness increased due to averted HIV infections. Although PEP drugs were the largest contributor to budget impact, the highest total costs were attributable to ART provision (∼84% of total; figure 2).

## Discussion

In this analysis, we evaluated the health and economic impact of providing online PrEP and PEP in western Kenya. We found that low coverage of online PrEP and PEP (1%) had a substantial effect on HIV incidence and was cost-effective from the Kenya Ministry of Health perspective. Online PEP was responsible for most intervention health benefits, likely due to high observed demand for PEP compared with PrEP from the ePrEP Kenya pilot study data used for model parameterisation. Further, online PrEP continuation and PEP-to-PrEP transition were low in the pilot study, with some clients with ongoing HIV risk opting for repeat PEP rather than PrEP. These findings are consistent with the PrEP literature, which shows low uptake and continuation of PrEP in both facility and community settings, despite considerable efforts to expand PrEP coverage^31^^,^^32^ One reason might be that PrEP uptake relies on anticipating potential future risks and engaging in proactive prevention, whereas PEP is sought reactively after a risk has occurred. Motivation for seeking HIV prevention could be higher after potential HIV exposure compared with future hypothetical risks. Additionally, the cost-effectiveness of PrEP depends on its alignment with HIV risk, which could be more difficult for individuals to predict, whereas PEP use is, by definition, aligned with potential HIV exposure. Our findings are consistent with a previous modelling analysis that found that community-based PEP provision was cost-effective.^7^ Further research is needed to evaluate effects of repeat PEP use and identify contexts in which it might be more appropriate than PrEP. Our results suggest that repeat PEP use could be beneficial to a substantial minority of clients, particularly women aged 25 years or older.

Online PrEP and PEP remained cost-effective from the Kenya Ministry of Health perspective across sensitivity analyses. In PEPFAR funding reduction scenarios, online PrEP and PEP became more cost-effective due to higher background HIV incidence and lower ART coverage; assuming PEPFAR disruptions to both HIV testing and ART resulted in the intervention being cost-saving and averting substantially more HIV burden than the main scenario. Introduction of low-cost long-acting monthly oral PrEP increased intervention health benefits and cost-effectiveness. However, as MK-8527 is not yet commercially available, we varied the price widely—as a result, cost-effectiveness of long-acting monthly oral PrEP based on MK-8527 is predicated on assumed price data only. Anticipated long-acting formulations can overcome barriers associated with daily pill taking including stigma and adherence issues. Studies have shown a strong preference for long-acting PrEP over daily oral pills in both the general and key populations (including sex workers and men who have sex with men) in eastern and southern Africa.^33^ Online pharmacies might also be well suited to deliver injectable PrEP, although service delivery costs would likely be higher due to the need for additional consumables and trained personnel to administer injections. However, a survey among potential online PrEP clients found higher willingness to pay for injectable compared to oral PrEP.^14^

Telehealth is a promising strategy to increase biomedical prevention coverage in eastern and southern Africa. Online PrEP and PEP can provide greater convenience, privacy, and autonomy compared to clinic-based services; long operating hours and weekend availability make online pharmacies well suited for PEP delivery, which requires timely provision to maximise effectiveness. Online pharmacies are rapidly growing in eastern and southern Africa and reach clients seeking discreet access to sexual health products, such as emergency contraception and HIV self-testing, including those who might be at risk for HIV. The ePrEP Kenya pilot study found that clients had sexual behaviours associated with HIV risk, indicating that the intervention reached those at substantial HIV risk.

Online PrEP and PEP has two important caveats. The first is that it largely reaches individuals at higher socioeconomic status with computer or smartphone literacy and internet access. Although the online PrEP and PEP platform can expand PrEP and PEP coverage to Kenya’s growing middle class, other community-based modalities are needed to serve individuals with lower socioeconomic status. However, we found that the intervention can attain cost-effective reductions on HIV burden with low population coverage and, therefore, could be used alongside other strategies to optimise biomedical prevention coverage. The second caveat is that online PrEP and PEP is only cost-effective when delivered through a private–public partnership in which the Kenya Ministry of Health pays for drugs and supply chain costs while remaining costs are borne by the online pharmacy and clients. In a full theoretical payer perspective, the intervention exceeded the cost-effectiveness threshold. However, we observed high demand for online PrEP and PEP in the ePrEP Kenya pilot study, in which clients were charged for HIV self-testing and delivery of testing and drugs. Additionally, MYDAWA has continued online PrEP and PEP provision outside of the pilot, with no reductions in client volume after implementing a $6·18 per-visit service fee (appendix 1 pp 11–12). This client fee is consistent with client-reported willingness to pay in the pilot study and another large survey^14^ (N=772) among individuals with HIV risk reporting interest in online PrEP and PEP recruited via MYDAWA’s website. With the changing global HIV funding landscape, innovative strategies that maximise shrinking health budgets to effectively deliver treatment and prevention are needed. Leveraging private retailers, including online pharmacies, can reach clients who are willing to pay for greater discretion and convenience while freeing up funds for higher cost community-based strategies to reach those at lower socioeconomic status. These partnerships can also benefit online pharmacies by expanding their customer base to clients who might purchase other products. However, additional studies outside of Kenya are needed to understand the uptake and performance of online PrEP and PEP in other countries in eastern and southern Africa. Financial and market analyses are also warranted to assess feasibility, demand, and financial costs in diverse contexts. Further, the health and cost effects of online PrEP and PEP are contingent on the scale reached; if demand is lower than projected, health benefits will likely be smaller and relative costs higher per client since fixed costs would be spread over a smaller client volume.

Our analysis has several limitations. We used data from a single pilot conducted in Nairobi and Mombasa; our results might not be generalisable outside urban areas of Kenya. Additionally, data from this single study might not be representative of online PrEP and PEP use patterns. Similar to previous modelling analyses,^8^ our results are sensitive to assumptions regarding PrEP and PEP uptake among those at substantial HIV risk. However, results from brick-and-mortar pharmacy-based PrEP and PEP studies in Kenya show similar sexual behaviour and high demand for PEP relative to PrEP among clients.^34^ Additionally, a previous study from eastern Africa found that making biomedical prevention widely available results in substantial population-level HIV incidence reduction despite low coverage, suggesting that individuals aligned PrEP use with periods of HIV risk.^35^ Our model only simulates heterosexual HIV transmission; therefore, we cannot evaluate online PrEP and PEP’s effect on men who have sex with men (MSM) or people who inject drugs. Indeed, the ePrEP Kenya pilot study showed that 47 (28%) of 227 PrEP users were MSM, but only 28 (3%) of 1688 PEP users reported being MSM;^19^ our analysis likely underestimates the intervention’s health effect and cost-effectiveness by not including this subgroup. Further, we do not have empirical data on online PrEP adherence; therefore, we used estimates from PrEP implementation studies.^19^ However, results were robust to sensitivity analyses. Further, findings were likely driven by PEP assumptions since it had substantially higher coverage than PrEP. Additionally, most clients accessing online PEP in the pilot study reported potential HIV exposure through consensual condomless sex;^12^ few reported sexual assault or occupational exposure, which has historically been the focus of clinic-based PEP provision in eastern and southern Africa. Therefore, other PEP delivery modalities remain important for these indications.

Strengths of this analysis include use of a network model and assessing uncertainty across 100 parameter sets. To our knowledge, this is the first modelling analysis to evaluate PEP scale-up by use of monthly time steps to align with HIV risk exposure and empirical data on PEP use stratified by age and sex from a real-world implementation study.

Overall, we found that PrEP and PEP provision via online pharmacies in Kenya can result in substantial HIV incidence reductions at low population coverage and is cost-effective from the Kenya Ministry of Health perspective. Our findings can inform policy decisions regarding scale-up of biomedical prevention in an era of shrinking donor funding.

## Equitable Partnership Declaration

## Data sharing

Data used for the modelling can be found in appendix 1 (pp 3–30). EMOD-HIV is open-source and publicly available at https://docs.idmod.org/projects/emod-hiv/en/2.20_a/.

## Declaration of interests

DK reports consulting fees from University College London and Massachusetts General Hospital. KN reports receiving support to attend International AIDS Society (IAS) conferences and meetings and is a member of the Executive Board and President-Elect of the IAS. All other authors declare no competing interests.
