# Supplementary material for: Cost-effectiveness of providing pre-exposure and post-exposure prophylaxis for the prevention of HIV via online pharmacies in western Kenya: a modelling study
Source: Lancet Glob Health. 2026 Aug 7;14(9):None. doi: 10.1016/j.langlo.2026.103977 (PMC13541651; doi:10.1016/j.langlo.2026.103977)
Supplement: Supplementary file 1 [file mmc1.pdf]

THE LANCET
Global Health

Supplementary appendix 1

This appendix formed part of the original submission and has been peer reviewed.
We post it as supplied by the authors.

Supplement to: Malhotra A, Patel N, Kaftan D, et al. Cost-effectiveness of providing pre-exposure and post-exposure prophylaxis for the prevention of HIV via online pharmacies in western Kenya: a modelling study. *Lancet Glob Health* 2026. https://doi.org/10.1016/j.langlo.2026.103977

Supplemental Appendix accompanying the manuscript: "Health and economic impact of providing pre- and post-exposure prophylaxis via online pharmacy in Kenya"

Akash Malhotra, Nishali Patel, David Kaftan, Yilin Chen, Cory Arrouzet, Arden Saravis, Catherine Kiptinness, Tabitha Kareithi, Kenneth Ngure, Katrina F. Ortblad, Monisha Sharma

Contents

Section 1 | Interventions and Analysis Design **3**
1.1 Intervention design 3
Table S1a. Target populations 3
Table S1b: Probability of up-taking PrEP or PEP among eligible subgroups 3
Table S1c. PrEP uptake in the model compared to pilot study 4
Table S1d. PEP uptake in the model compared to pilot study 4
Table S1e. Assumed RDT sensitivity 5
Figure S1. Schematic depicting model implementation of e-PEP and PrEP delivery 7
1.2 Cost approach and calculations 7
Table S2. Cost parameter calculations ¥ 9
1.3 Client volume and cost data after pilot completion 11

Section 2 | Model Calibration and Key Parameters **12**
2.1 Model calibration to HIV parameters 12
Table S3a. HIV prevalence in counties of the former Nyanza province, by age and sex¥ 12
Table S3b. HIV prevalence among men and women ages 15-49, by county and gender¥ 12
Table S4. Number of people on ART by county, sex, and age group¥ 14
Table S5. Circumcision status quo by county, age group, and year ¥ 16
Figure S3. Model fit to age-specific and overall prevalence from population-based surveys by sex 18
Table S6. Key model parameters. 22
Table S7. Disability weights for estimating disability-adjusted life-years averted 25
2.2 Model calibration to population parameters 26
Table S8. Population size by gender, county, and age group in 2009¥ 26
Table S9a. Age-specific population fertility rates in Kenya 1950-2049¥ 28
Table S9b. Age-specific mortality rates among in Kenya 1950-2049 by gender ¥ 29
Figure S4. Model predicted population growth by sex 30

Section 3 | Additional Results **31**
3.1 Sensitivity analyses 31
Table S10: Sensitivity analyses: higher MOH costs and PEPFAR interruptions 31
Table S11a. Uptake for individuals aged 15-49 with any condomless sex act with a non-marital partner 31
Table S11b. Health and economic impact of broad PEP and PrEP targeting 32
3.2 Replacing daily oral PrEP and PEP with a long acting monthly oral pill 32
Table S12. Incremental cost-effectiveness ratio using MK-8527 at varying costs 32
3.3 Additional Results 32
Table S13a. HIV infections averted among ages 15-49 in each scenario (2026 - 2036) 33
Table S13b. HIV deaths averted among ages 15-49 in each scenario (2026 - 2061) 33
Table S13c. Distribution of PEP initiations per person year by age, sex, and partnership type 34
Table S13d. HIV infections averted by age and sex (2026-2036) 34
Figure S5. Total 35 year undiscounted costs (in $M) by category for each modeled scenario 35

Figure S6. Cost difference by component relative to baseline over 35 years 36
Figure S7. Total PrEP and PEP doses by scenario (2026-2036) 37
Figure S8. Cost-effectiveness acceptability curve 37
References 38

Section 1 | Interventions and Analysis Design

1.1 Intervention design

Summary of populations targeted and implementation scenarios

Table S1a. Target populations

Target population	Description
People with multiple concurrent partners	Individuals with 2 concurrent partners simultaneously.
People with more than 1 partner in the last 3 months	Individuals with 2 or more partners in the past 3 months.
Men and women in serodiscordant relationships and with multiple concurrent partners	Serodiscordant couples; persons aware their partner has HIV and is not on ART* and who have 2 concurrent partners.
Men and women in serodiscordant relationships and with more than 1 partner in the last 3 months	Serodiscordant couples; individuals who are aware their partner has HIV and is not on ART*. Additionally, these people have 2 or more partners in the last 3 months.

*We assume 50% of individuals with diagnosed HIV in a partnership disclose their status to their partners.

Table S1b: Probability of up-taking PrEP or PEP among eligible subgroups

	PrEP	PEP
AGYW with multiple concurrent partners	0.38%	0.60%
AGYW with >1 partners in the last 3 months	0.70%	1.06%
ABYM with multiple concurrent partners	0.90%	2.40%
ABYM with >1 partners in the last 3 months	1.28%	2.24%
Women with multiple concurrent partners	1.67%	3.79%
Women with multiple concurrent partners and in serodiscordant relationships	3.25%	-
Women with >1 partners in the last 3 months	2.90%	5.19%
Women with >1 partners in the last 3 months and in serodiscordant relationships	5.50%	-
Men with multiple concurrent partners	1.15%	3.50%
Men with multiple concurrent partners and in serodiscordant relationships	1.75%	-
Men with >1 partners in the last 3 months	1.50%	2.89%
Men with >1 partners in the last 3 months and in serodiscordant relationships	2.80%	-

Table S1c. PrEP uptake in the model compared to pilot study

**Population**	**Pilot uptake**	**Model uptake**
ABYM with multiple concurrent partners	10%	10%
ABYM with multiple partners in the last 3 months	8%	8%
AGYW with multiple concurrent partners	7%	6%
AGYW with multiple partners in the last 3 months	6%	6%
Men with multiple concurrent partners	28%	27%
Men with multiple concurrent partners and in serodiscordant relationships	3%	3%
Men with multiple partners in the last 3 months	13%	12%
Men with multiple partners in the last 3 months and in serodiscordant relationships	2%	4%
Women with multiple concurrent partners	10%	11%
Women with multiple concurrent partners and in serodiscordant relationships	2%	2%
Women with multiple partners in the last 3 months	10%	9%
Women with multiple partners in the last 3 months and in serodiscordant relationships	1%	2%

Table S1d. PEP uptake in the model compared to pilot study

**Population**	**Pilot uptake**	**Model uptake**
AGYW with multiple concurrent partners	4%	4%
AGYW with multiple partners in the last 3 months	7%	8%
ABYM with multiple concurrent partners	8%	8%
ABYM with multiple partners in the last 3 months	9%	9%
Women with multiple concurrent partners	10%	10%
Women with multiple partners in the last 3 months	15%	14%
Men with multiple concurrent partners	24%	25%
Men with multiple partners in the last 3 months	22%	21%
**Relative uptake of PEP vs PrEP**		
**All populations**	6.8x	6.4x

Definitions

Initiation: Start of a PrEP or PEP course by an eligible individual following screening and eligibility assessment.

PrEP or PEP use: Periods during which an individual is receiving PrEP or PEP , for PrEP this represents both initiation and continuation.

Coverage: total number of person-months on PrEP or PEP, divided by total population person-months during the analysis period.

Background HIV testing

Background HIV testing was assumed to be a provider-administered Ab rapid diagnostic test (RDT). Test sensitivity increased from 0.0% in the first 19 days of infection to 99.9% after 120 days of infection. These values were derived from a review of empiric data in the literature, including data from *Taylor et al* on the cumulative probability of a negative test result for a third- & fourth-generation HIV test at various time points in an HIV-positive individual[1]. We first calculated the inverse of cumulative probability to obtain the cumulative probability of a positive test result. To adjust for RDTs (as these were excluded from Taylor et al. 2015), we shifted the inverse cumulative probability 10 days forward. Finally, we made an adjustment to Ab RDT at day 45 by using mean of day 30 and 60 (0.865 vs. 0.950). See Cox, Wu et al 2024 for additional information on test sensitivity parameterization in this model[2].

Table S1e. Assumed RDT sensitivity

**Day from exposure:**	0-18.9	19-21.9	22-29.9	30-44.9	45-59.9	60-89.9	90-119.9	120+
**Ab RDT sensitivity:**	00.0%	20.0%	37.0%	78.0%	86.5%	95.0%	97.0%	99.9%

PEP provision

PEP was modeled as a 1-month regimen with 95% efficacy, initiated by a proportion of those in the target population with a recent non-marital condomless sex act and not known to be HIV-positive. Clients initiated PEP after the exposure (26 hours median time from exposure to PEP initiation reported in the pilot study)[3]. After completing the 1-month PEP course, individuals had a 50% probability of returning for follow-up HIV testing. Those who tested positive were referred to ART, those who tested HIV-negative could transition to PrEP at the rates observed in the pilot if they met eligibility criteria.

In EMOD-HIV, PEP is implemented using the ControlledVaccine intervention class. When a PEP-eligible coital act occurs within a monthly time step, it can trigger PEP distribution, based on the modelled person's probability of seeking PEP which we set to be dependent on individual's

demographics and sexual behavior. PEP distribution reduces the individual's likelihood of acquiring HIV within the time step. It acts as an acquisition-blocking intervention that prevents the person from getting infected during the timestep when PEP is distributed rather than curing an infection after it occurs.

PrEP provision

Individuals without known HIV and who tested HIV-negative initiated PrEP (30 day supply with 75% efficacy). After 30 days, individuals had a 30% probability returning for a PrEP refill; those testing HIV-negative and had at least one sexual partner received PrEP (3-monthly supply). Individuals who tested HIV-positive via the PrEP cascade were assumed to link to ART.

Figure S1. Schematic depicting model implementation of e-PEP and PrEP delivery

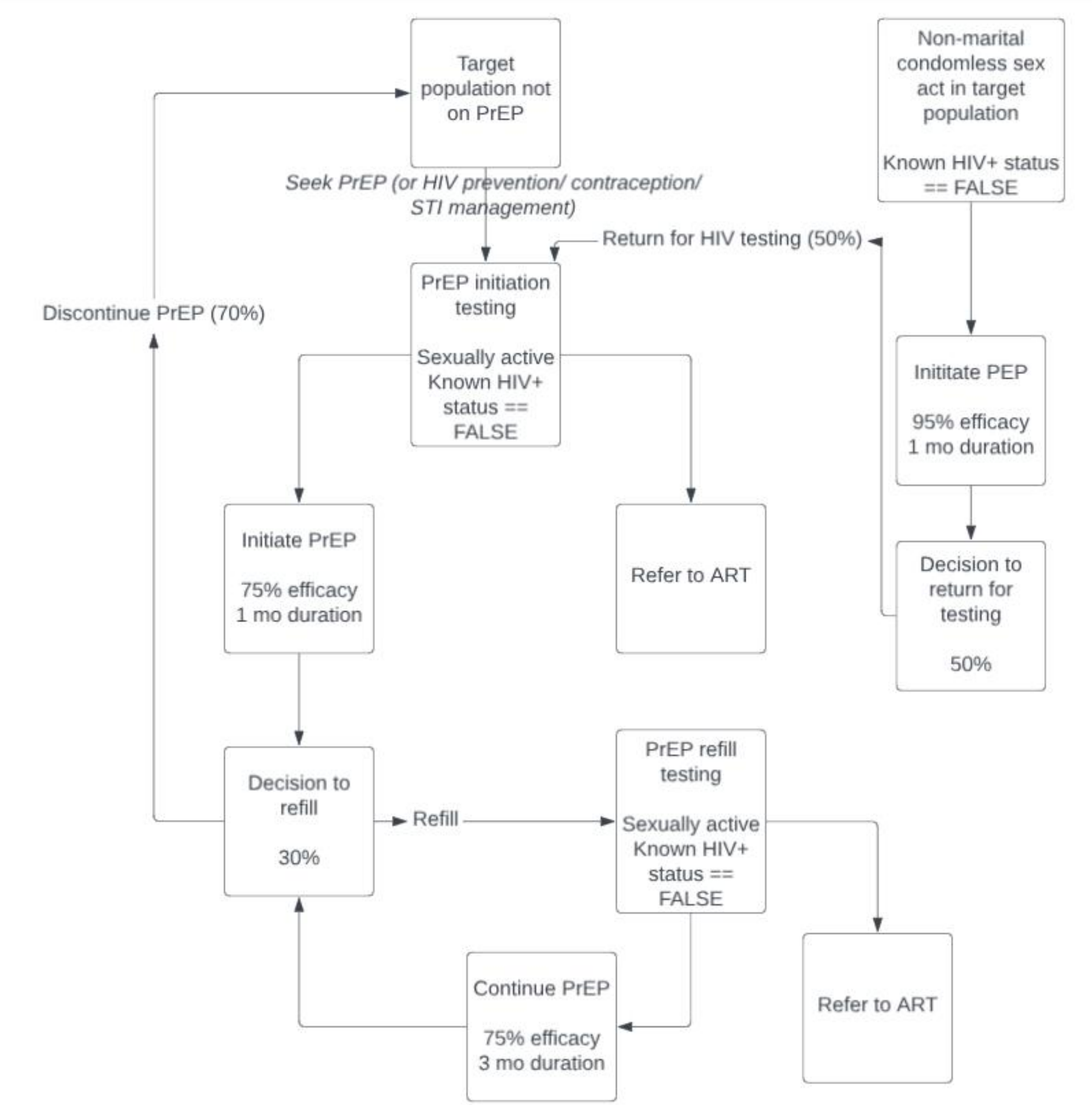

1.2 Cost approach and calculations

Service delivery model: We assume a public/private partnership cost-sharing model for ePharmacy-based PrEP and PEP delivery with pharmacy costs partially offset by client fees. We assumed the Ministry of Health (MoH) incurred costs for PrEP and PEP drugs, ART, HIV-related hospitalizations, and HIV-related deaths. All other service delivery costs including pharmacy staff salaries and benefits, demand generation, clinical consultations, microplanning and training, user support, vehicles, equipment, fuel, and utilities were incurred by the ePharmacy.

Detailed breakdown of these costs from a microcosting of the pilot study have been reported (Chen et al., 2025)[4]. Clients are assumed to pay for HIV self-test kits ($4.05 per kit) and a $1 delivery fee for HIVST or drug delivery. In a willingness-to-pay study (Montano et al., 2025), current and potential ePharmacy clients reported willingness to pay an additional $7 for a bundled HIV prevention package (excluding delivery and HIV testing costs)[5].

Perspective for economic evaluation: Cost-effectiveness was evaluated from the Ministry of Health perspective. Costs borne by the epharmacy and clients were excluded from the economic evaluation.

Cost sources and adjustments: Cost estimates were derived from the published literature. Estimates for some components (e.g., RDT costs) varied widely among published studies, and in these cases, we prioritized studies based on geographic relevance, recency, sample size, and methodology. For all studies conducted prior to 2017, we adjusted for inflation to 2021 US$ using World Bank inflation estimates[6]. For all studies which collected data in a country besides Kenya, we adjusted the estimates by taking a ratio of the GDP per capita of the two countries to approximate the differences in purchasing power parity between the two settings, since PPP or health-specific CPIs were not reliably available in these settings[6,7]. We discounted costs and health outcomes by 3% annually.[8]

Table S2. Cost parameter calculations [¥]

Cost Parameter	Estimate (US$)	Year †	Data source	Calculation details, notes
Annual health care costs (among those not on ART)				
HIV-positive CD4 < 200	110.30	2021	Eaton[9]	Adjusted for inflation and GDP/capita ratio using this approach:<br>Step 1: Adjust South Africa value in 2012 US$ for inflation to be in 2021 US$ ($374.08 = $167*2.24)<br>● Cost of health care use, CD4 count <200 cells per µL, not in HIV care (per person-year) in South Africa= $167<br>● US$ Inflation Rate between time of costing (2012) and 2021: 2.24 =4.7/2.1<br>Step 2: Adjust South Africa 2021 US$ value by multiplying by the Kenya GDP/cap ratio ($374.08*0.295)<br>● South Africa 2021 GDP per capita in US$ = 7,055; Kenya US$ = 2,082<br>● Kenya GDP/ ZA GDP ratio adjustment: (2,082/7,055)= 0.295
HIV-positive CD4 200 - 349	30.38	2021	Eaton[9]	Adjusted for inflation and GDP/capita ratio (see steps above)
HIV-positive CD4 > 350	8.59	2021	Eaton[9]	Adjusted for inflation and GDP/capita ratio (see steps above)
End of life care	105.68	2021	Eaton[9]	Adjusted for inflation and GDP/capita ratio (see steps above)
Annual ART provision costs	140.89	2021 / 2016	Phillips[10], Larson[11], Global Fund[12]	Includes 20% mark up on ART drug costs to account for supply chain (2021 US$). ART delivery costs are from an in-country micro-costing study and include lab tests and staff encounters (2016 US$).<br>Step 1: Calculate annual ART cost accounting for 20% supply chain cost mark-up ($57.89= $48.24*1.2)<br>● Monthly ART cost (per pack, 30 for Dolutegravir/Lamivudine/Tenofovir 50/300/300mg tablet)= $4.02<br>● Annual ART cost= $48.24= $4.02*12<br>● 20% supply chain cost mark-up= *1.2

				Step 2: Calculate total cost of ART delivery ($83=45+38) and add to Step 1<br>● $45 lab costs<br>● $38 staff encounters
Cost of 30 days of PrEP medication	4.92	2025	Chen[4]	-
Cost of 30 days of PEP medication	6.94	2025	Chen[4]	-
Facility-based HIV-positive test	3.68	2017	Meisner[13]	Data from micro-costing study. Inputs include screening test kit, other supply costs, and personnel costs.
Facility-based HIV-negative test	2.64	2017	Meisner[13]	Data from micro-costing study. Inputs include screening test kit, other supply costs, and personnel costs.
Referral if the HIV test for PrEP initiation is positive	$0.31	2016 / 2022	Meisner[13], Mangale[14]	-

¥ PrEP: pre-exposure prophylaxis, PEP: post-exposure prophylaxis ART: antiretroviral therapy. All HIV test, ART provision costs include personnel, overhead, and supplies.
† Inflation adjustment using data from the World Bank (% inflation, consumer prices), applied only if reference provides cost estimates for years prior to 2016.

1.3 Client volume and cost data after pilot completion

Based on project volume data obtained from MYDAWA on September 18, 2025, client volumes were stable after the pilot ended and MYDAWA continued services and implemented additional client fees[15]. A per-visit service fee of $6.18 is charged each time a client seeks PrEP or PEP services through MYDAWA, covering clinical consultation, routine follow-ups, and delivery of medicines and test kits. PrEP and PEP drugs and HIV test kits remain free of charge.

Figure S2.1 Monthly number of clients accessing MYDAWA's online PrEP and PEP services

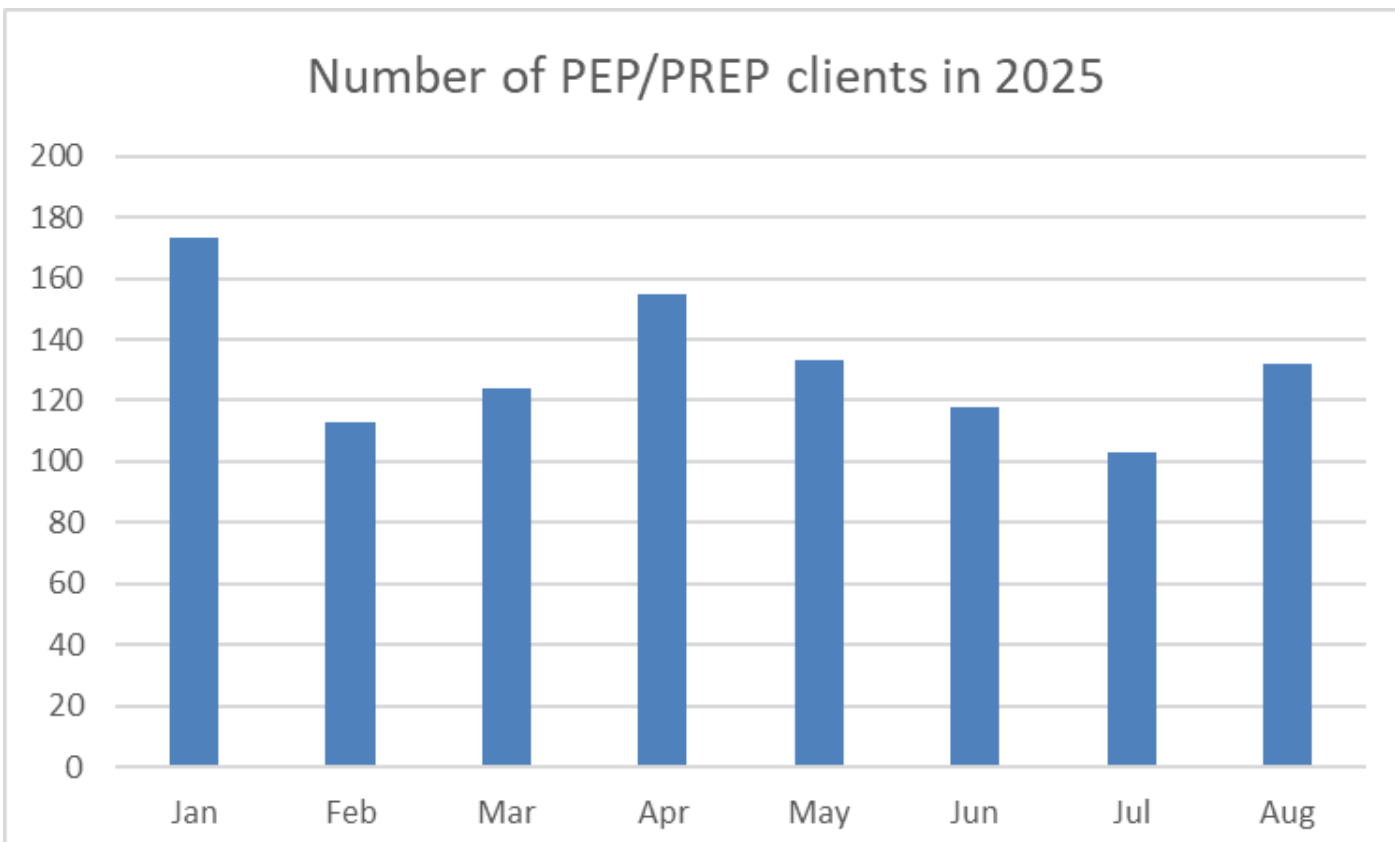

Figure S2.2 Number of MYDAWA online PrEP vs PEP clients

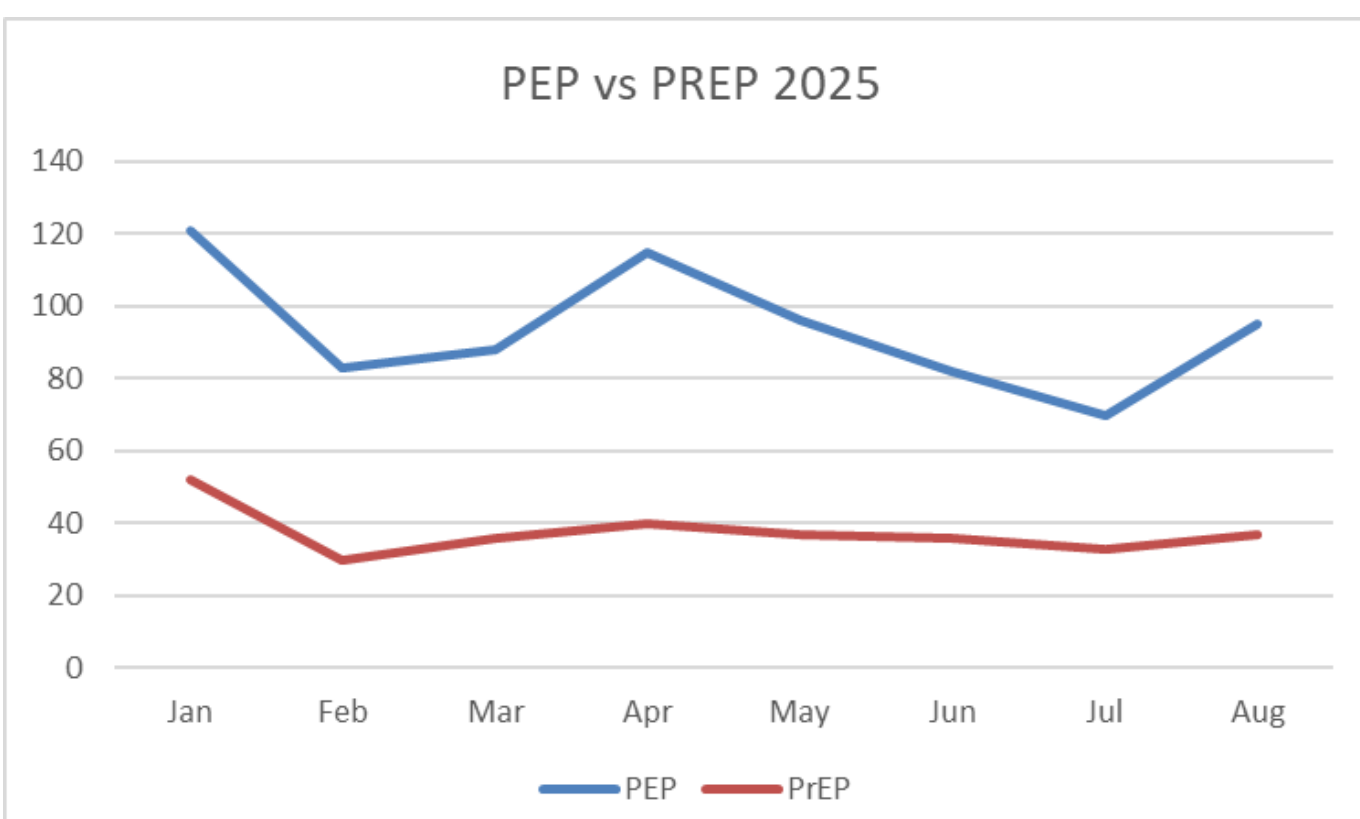

Figure S2.3 Number of online PrEP refills via MYDAWA

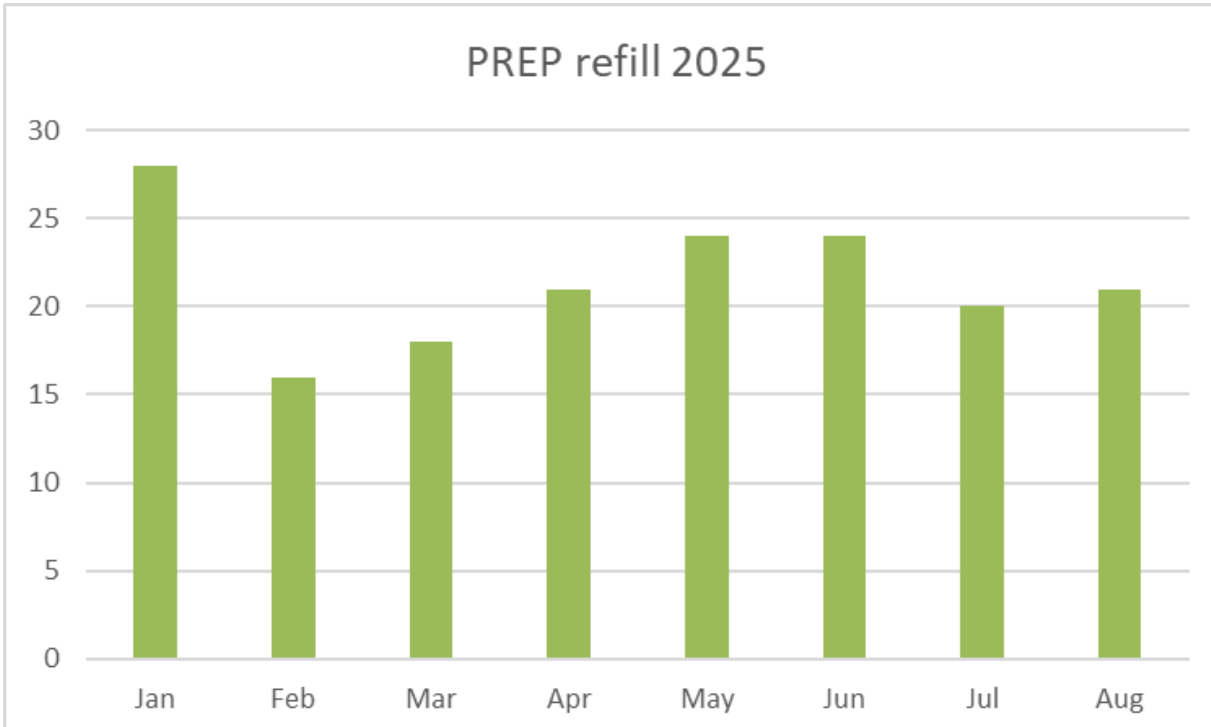

Section 2 | Model Calibration and Key Parameters

2.1 Model calibration to HIV parameters

<u>Model calibration</u>

Table S3a. HIV prevalence in counties of the former Nyanza province, by age and sex[¥]

Age group	2003		2007		2008		2012		2018	
	Men	Women	Men	Women	Men	Women	Men	Women	Men	Women
15 - 19	0.0015	0.0459	0.0121	0.0773	0.0184	0.1078	0.0151	0.0486	0.0025	0.0294
20 - 24	0.0562	0.2997	0.0257	0.2056	0.0578	0.1201	0.0289	0.1411	0.0216	0.0910
25 - 29	0.2429	0.2301	0.1956	0.2454	0.2450	0.2228	0.2107	0.2454	0.0705	0.2334
30 - 34	0.1840	0.1632	0.2578	0.2576	0.1530	0.2593	0.2392	0.2047	0.0911	0.2696
35- 39	0.2064	0.1838	0.2384	0.2227	0.2275	0.2259	0.1955	0.2811	0.1675	0.2705
40 - 44	0.2533	0.3500	0.2024	0.1799	0.2501	0.0927	0.3132	0.1694	0.2075	0.2765
45 - 49	0.1624	0.1651	0.2103	0.1291	0.1331	0.1716	0.1623	0.2287	0.2796	0.1991
15 - 49	0.1160	0.1830	0.1140	0.1760	0.1140	0.1600	0.1340	0.1760	0.0826	0.1667

[¥]**Sources:** Kenya Demographic and Health Surveys, 2003 & 2008; Kenya AIDS Indicator Surveys, 2007 & 2012; Kenya Population-based HIV Impact Assessment 2018. The counties of the former Nyanza province are Homa Bay, Kisii, Kisumu, Migori, Nyamira, and Siaya.

Table S3b. HIV prevalence among men and women ages 15-49, by county and gender[¥]

County	2003		2007		2008		2012		2018	
	Men	Women	Men	Women	Men	Women	Men	Women	Men	Women

Homa Bay	0.1097	0.2458	0.2514	0.3259	0.1737	0.2524	0.2217	0.2787	0.1279	0.2532
Kisii	0.0114	0.0853	0.0445	0.0693	0.0330	0.0573	0.0346	0.0368	0.0458	0.0684
Kisumu	0.1663	0.1914	0.1139	0.1847	0.1109	0.1810	0.1940	0.2022	0.0960	0.2096
Migori	0.1804	0.1860	0.1685	0.2181	0.1923	0.2228	0.1435	0.1925	0.0706	0.1758
Nyamira	0.0029	0.0742	-	-	0.0234	0.0544	0.0419	0.1045	0.0247	0.0432
Siaya	0.1824	0.2424	0.1445	0.2130	0.1526	0.1921	0.2596	0.2990	0.0961	0.1905

¥**Sources:** Kenya Demographic and Health Surveys, 2003 & 2008; Kenya AIDS Indicator Surveys, 2007 & 2012; Kenya Population-based HIV Impact Assessment 2018.

Table S4. Number of people on ART by county, sex, and age group¥

Gender	County	Age group	Year													
			2004	2005	2006	2007	2008	2009	2010	2011	2012	2013	2014	2015	2016	2017
Men	Homa Bay	0 - 14	-	-	-	-	-	-	-	-	-	-	2,945	3,583	4,109	4,192
		15 - 99	1,067	2,313	5,148	7,194	10,002	14,436	17,178	15,954	17,522	18,279	19,157	22,834	26,441	29,220
	Kisii	0 - 14	-	-	-	-	-	-	-	-	-	-	828	993	1,109	1,083
		15 - 99	-	-	-	-	-	-	-	2,972	-	-	4,614	5,451	6,604	7,169
	Kisumu	0 - 14	-	-	-	-	-	-	-	-	-	-	3,101	3,245	3,525	3,607
		15 - 99	945	2,047	4,557	6,368	8,853	12,779	15,206	14,122	15,511	16,180	21,216	24,550	28,082	31,021
	Migori	0 - 14											2,309	2,295	2,678	2,673
		15 - 99	711	1,541	3,430	4,793	6,664	9,619	11,446	10,630	11,675	12,179	13,929	15,165	17,438	18,455
	Nyamira	0 - 14	-	-	-	-	-	-	-	-	-	-	484	552	578	611
		15 - 99	-	-	-	-	-	-	-	1,362	-	-	2,120	2,585	3,142	3,474
	Siaya	0 - 14	-	-	-	-	-	-	-	-	-	-	2,645	2,950	3,017	3,197
		15 - 99	860	1,864	4,148	5,797	8,060	11,633	13,843	12,856	14,120	14,730	16,163	18,611	21,477	23,762
Women	Homa Bay	0 - 14	-	-	-	-	-	-	-	-	-	-	3,431	3,835	4,426	4,535
		15 - 99	1,359	2,944	6,551	9,155	12,522	17,202	21,798	31,454	35,182	38,819	40,118	49,956	57,286	61,811
	Kisii	0 - 14	-	-	-	-	-	-	-	-	-	-	906	1,079	1,200	1,146
		15 - 99	-	-	-	-	-	-	-	7,902	-	-	11,691	14,350	17,274	19,044
	Kisumu	0 - 14	-	-	-	-	-	-	-	-	-	-	3,241	3,393	3,810	3,831
		15 - 99	1,203	2,606	5,799	8,104	11,084	15,227	19,296	27,843	31,143	34,362	41,230	48,424	56,384	60,789
	Migori	0 - 14	-	-	-	-	-	-	-	-	-	-	2,526	2,448	2,868	2,884

		15 - 99	905	1,961	4,365	6,100	8,343	11,461	14,524	20,958	23,442	25,865	27,896	31,964	38,637	40,891
	Nyamira	0 - 14	-	-	-	-	-	-	-	-	-	-	506	567	601	622
		15 - 99	-	-	-	-	-	-	-	3,766	-	-	5,964	7,210	8,258	8,654
	Siaya	0 - 14	-	-	-	-	-	-	-	-	-	-	2,778	3,136	3,299	3,569
		15 - 99	1,095	2,372	5,279	7,377	10,090	13,862	17,566	25,347	28,351	31,281	33,911	39,853	44,892	48,808
Both	All	15-99	8,954	19,404	43,185	60,350	83,142	116,787	143,877	175,003	194,552	210,769	246,293	301,029	333,333	389,159

[¥]Source: Kenya Ministry of Health

Table S5. Circumcision status quo by county, age group, and year ¥

	Homa Bay			Kisii			Kisumu			Migori			Nyamira			Siaya		
Year	10-14	15-24	25-49	10-14	15-24	25-49	10-14	15-24	25-49	10-14	15-24	25-49	10-14	15-24	25-49	10-14	15-24	25-49
Pre-2008	0.249	0.249	0.249	0.948	0.948	0.948	0.322	0.322	0.322	0.410	0.410	0.410	0.965	0.965	0.965	0.252	0.252	0.252
2008	0.118	0.235	0.275	0.948	0.948	0.948	0.152	0.303	0.333	0.194	0.385	0.451	0.965	0.965	0.965	0.119	0.243	0.270
2009	0.122	0.240	0.283	0.948	0.948	0.948	0.178	0.315	0.339	0.199	0.388	0.463	0.965	0.965	0.965	0.127	0.250	0.277
2010	0.166	0.285	0.299	0.948	0.948	0.948	0.324	0.389	0.362	0.214	0.395	0.478	0.965	0.965	0.965	0.222	0.310	0.293
2011	0.226	0.355	0.313	0.948	0.948	0.948	0.450	0.479	0.385	0.280	0.423	0.486	0.965	0.965	0.965	0.293	0.375	0.304
2012	0.337	0.486	0.339	0.948	0.948	0.948	0.516	0.559	0.409	0.516	0.530	0.509	0.965	0.965	0.965	0.417	0.483	0.324
2013	0.368	0.565	0.361	0.948	0.948	0.948	0.564	0.634	0.436	0.587	0.614	0.528	0.965	0.965	0.965	0.435	0.549	0.340
2014	0.471	0.710	0.399	0.948	0.948	0.948	0.620	0.712	0.467	0.717	0.726	0.554	0.965	0.965	0.965	0.537	0.659	0.368
2015	0.506	0.811	0.438	0.948	0.948	0.948	0.672	0.790	0.502	0.742	0.813	0.580	0.965	0.965	0.965	0.562	0.739	0.395
2016	0.476	0.894	0.488	0.948	0.948	0.948	0.756	0.844	0.538	0.697	0.891	0.613	0.965	0.965	0.965	0.628	0.841	0.425
2017	0.489	0.937	0.537	0.948	0.948	0.948	0.863	0.889	0.572	0.680	0.949	0.648	0.965	0.965	0.965	0.759	0.917	0.457
2018	0.589	0.925	0.637	0.948	0.948	0.948	0.944	0.925	0.651	0.775	0.976	0.744	0.965	0.965	0.965	0.844	0.959	0.562
2019	0.741	0.904	0.678	NA	NA	NA	0.784	0.943	0.677	0.785	NA	0.775	NA	NA	NA	0.790	0.976	0.596
2020	0.802	0.901	0.717	NA	NA	NA	0.787	0.925	0.703	0.800	NA	0.808	NA	NA	NA	0.800	NA	0.633

2021	0.802	0.909	0.749	NA	NA	NA	0.836	0.913	0.727	NA	NA	0.836	NA	NA	NA	NA	NA	0.668
2022	NA	0.917	0.779	NA	NA	NA	0.804	0.947	0.750	NA	NA	0.865	NA	NA	NA	NA	NA	0.704
2023	NA	0.926	0.807	NA	NA	NA	0.804	NA	0.773	NA	NA	0.892	NA	NA	NA	NA	NA	0.738
2024	NA	0.935	0.833	NA	NA	NA	0.804	NA	0.795	NA	NA	0.918	NA	NA	NA	NA	NA	0.772
2025	NA	0.944	0.858	NA	NA	NA	0.804	NA	0.815	NA	NA	0.942	NA	NA	NA	NA	NA	0.803
2026	NA	0.949	0.879	NA	NA	NA	0.801	NA	0.832	NA	NA	0.961	NA	NA	NA	NA	NA	0.832
2027	NA	0.953	0.898	NA	NA	NA	0.801	NA	0.848	NA	NA	0.976	NA	NA	NA	NA	NA	0.976
2028	NA	0.955	0.915	NA	NA	NA	0.801	NA	0.862	NA	NA	NA	NA	NA	NA	NA	NA	NA
2029	NA	NA	0.931	NA	NA	NA	0.801	NA	0.874	NA	NA	NA	NA	NA	NA	NA	NA	NA
2030	NA	NA	0.945	NA	NA	NA	0.801	NA	0.885	NA	NA	NA	NA	NA	NA	NA	NA	NA
2031	NA	NA	0.955	NA	NA	NA	0.802	NA	0.897	NA	NA	NA	NA	NA	NA	NA	NA	NA
2032	NA	NA	NA	NA	NA	NA	NA	NA	0.907	NA	NA	NA	NA	NA	NA	NA	NA	NA
2033	NA	NA	NA	NA	NA	NA	NA	NA	0.917	NA	NA	NA	NA	NA	NA	NA	NA	NA
2034	NA	NA	NA	NA	NA	NA	NA	NA	0.924	NA	NA	NA	NA	NA	NA	NA	NA	NA
2035	NA	NA	NA	NA	NA	NA	NA	NA	0.931	NA	NA	NA	NA	NA	NA	NA	NA	NA

¥**Source:** Circumcision prevalence prior to 2008 is obtained from the Kenya Demographic and Health Survey, 2003. Prevalence of circumcision from 2008 onward combines prevalence of traditional male circumcision and voluntary medical male circumcision estimates obtained from the Decision-Makers' Program Planning Toolkit 2.

Figure S3. Model fit to age-specific and overall prevalence from population-based surveys by sex

* Blue curves refer to the LOESS line fitting to the 100 simulations; the error bars refer to the empirical estimates and 95% confidence intervals for HIV prevalence obtained from Kenya Demographic and Health Surveys and Kenya AIDS Indicator Surveys.

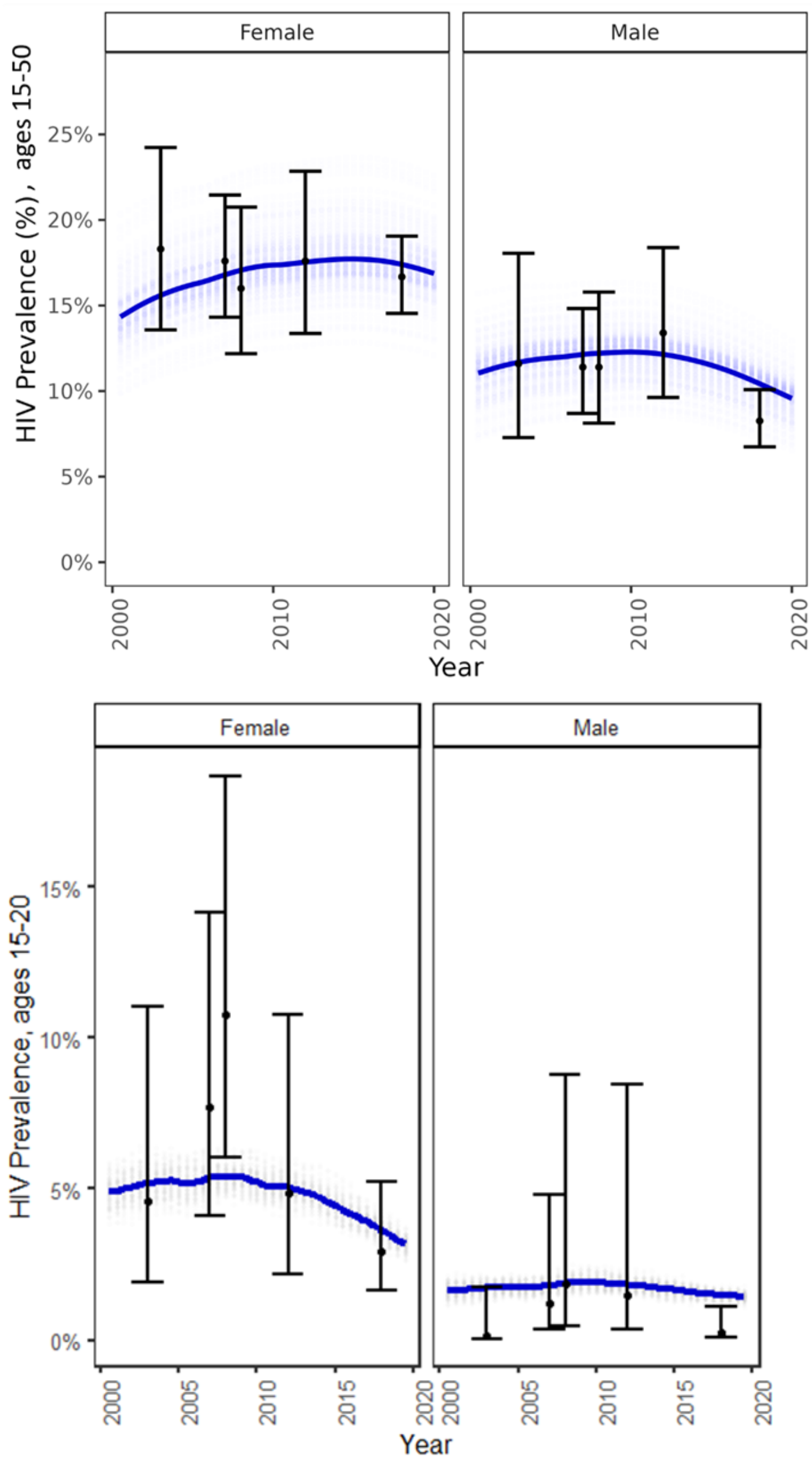

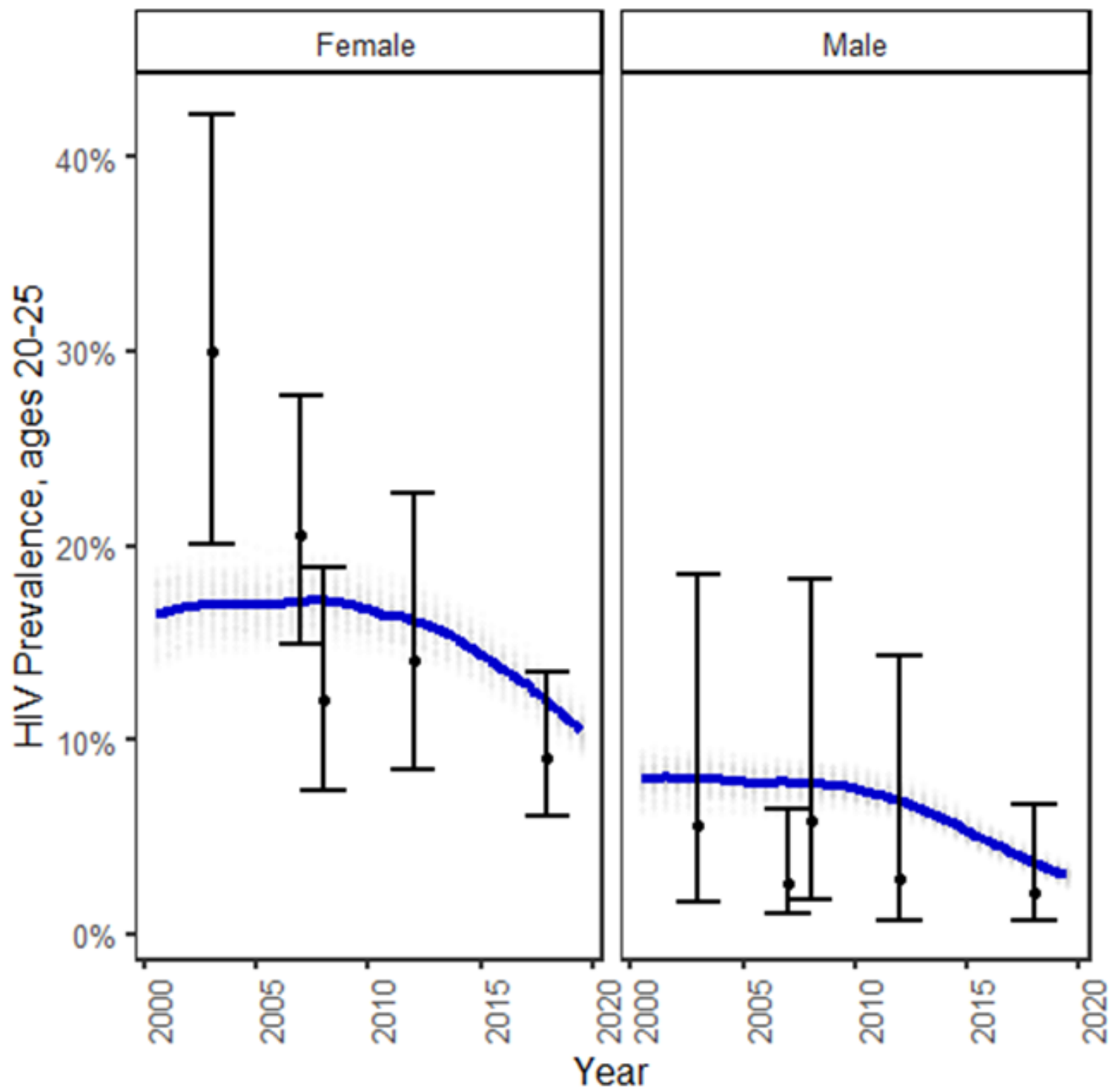
Female
Male
HIV Prevalence, ages 20-25
40%
30%
20%
10%
0%
2000
2005
2010
2015
2020
Year

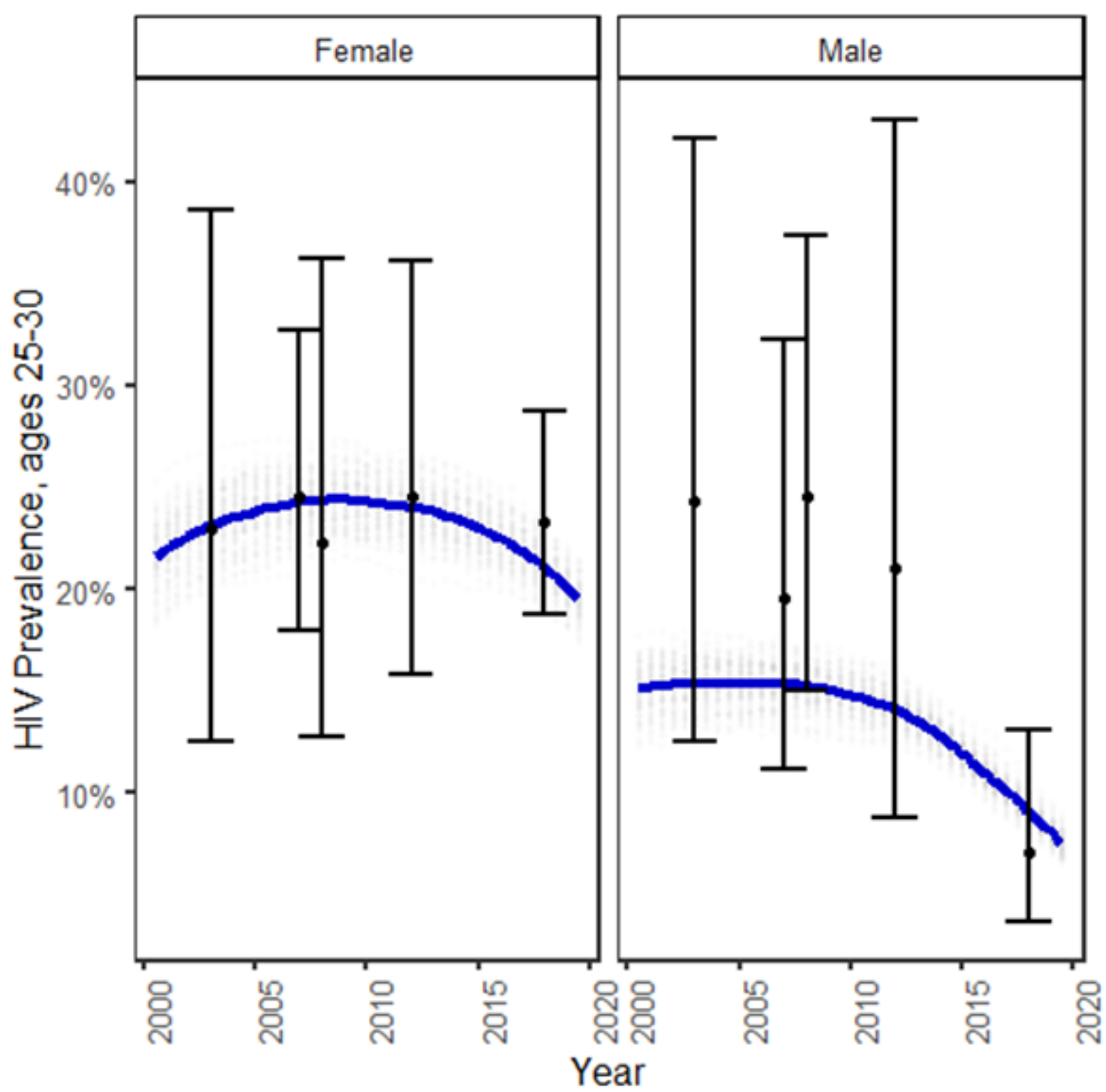
Female
Male
HIV Prevalence, ages 25-30
40%
30%
20%
10%
2000
2005
2010
2015
2020
Year

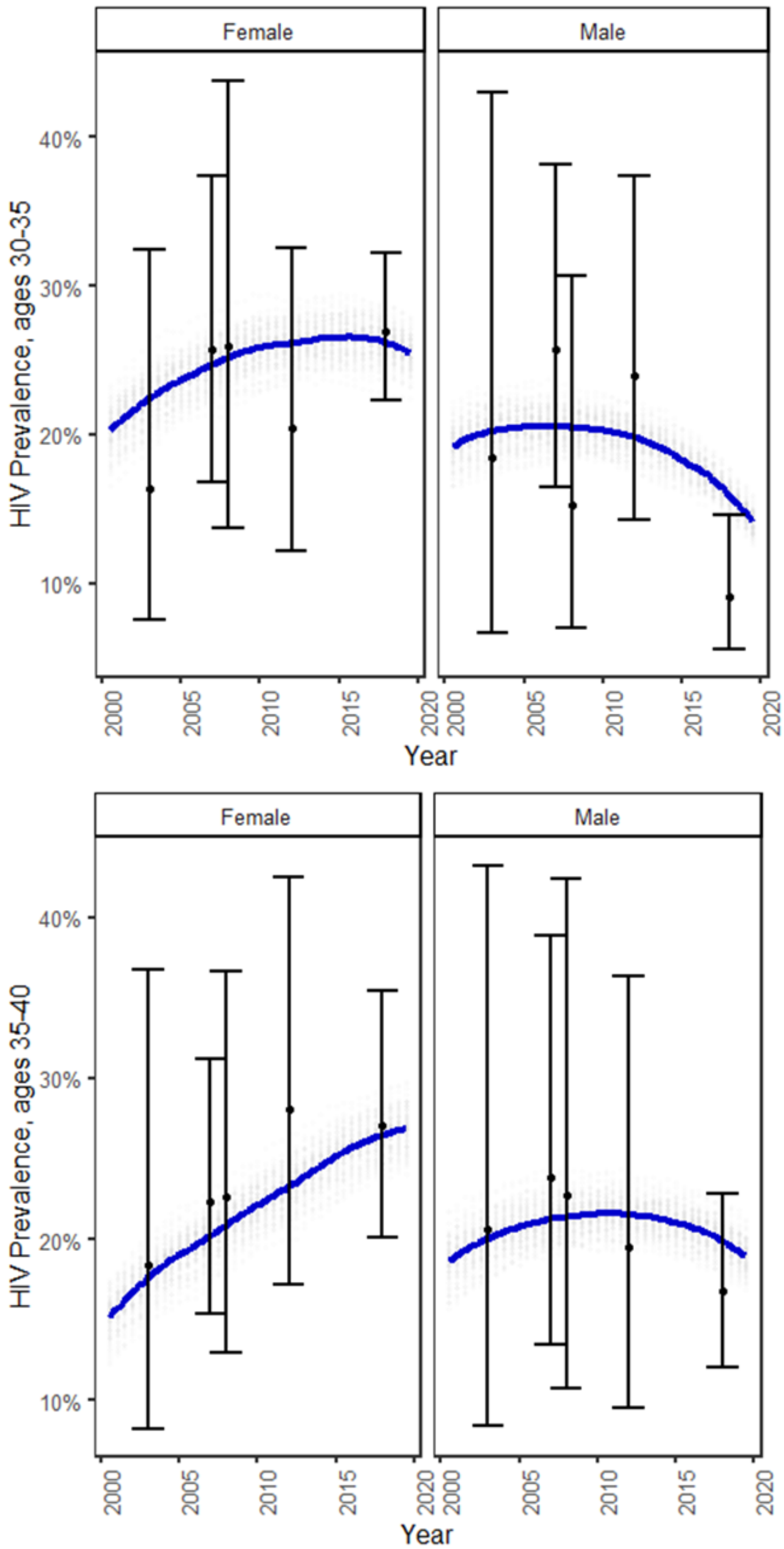

Female
Male
40%
30%
20%
10%
HIV Prevalence, ages 30-35
2000
2005
2010
2015
2020
Year
Female
Male
HIV Prevalence, ages 35-40
Year

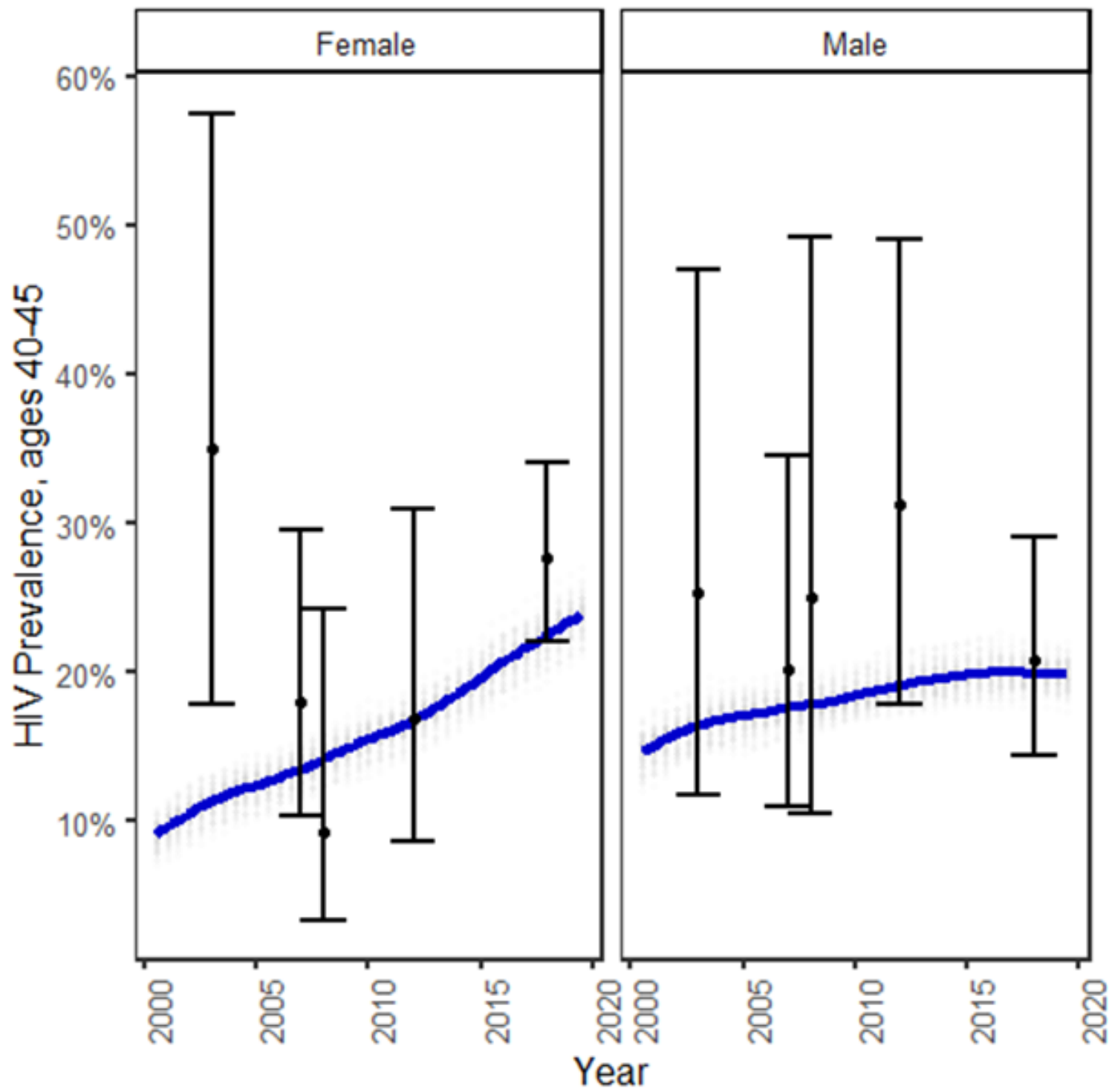

Female
Male
60%
50%
40%
30%
20%
10%
HIV Prevalence, ages 40-45
2000
2005
2010
2015
2020
Year

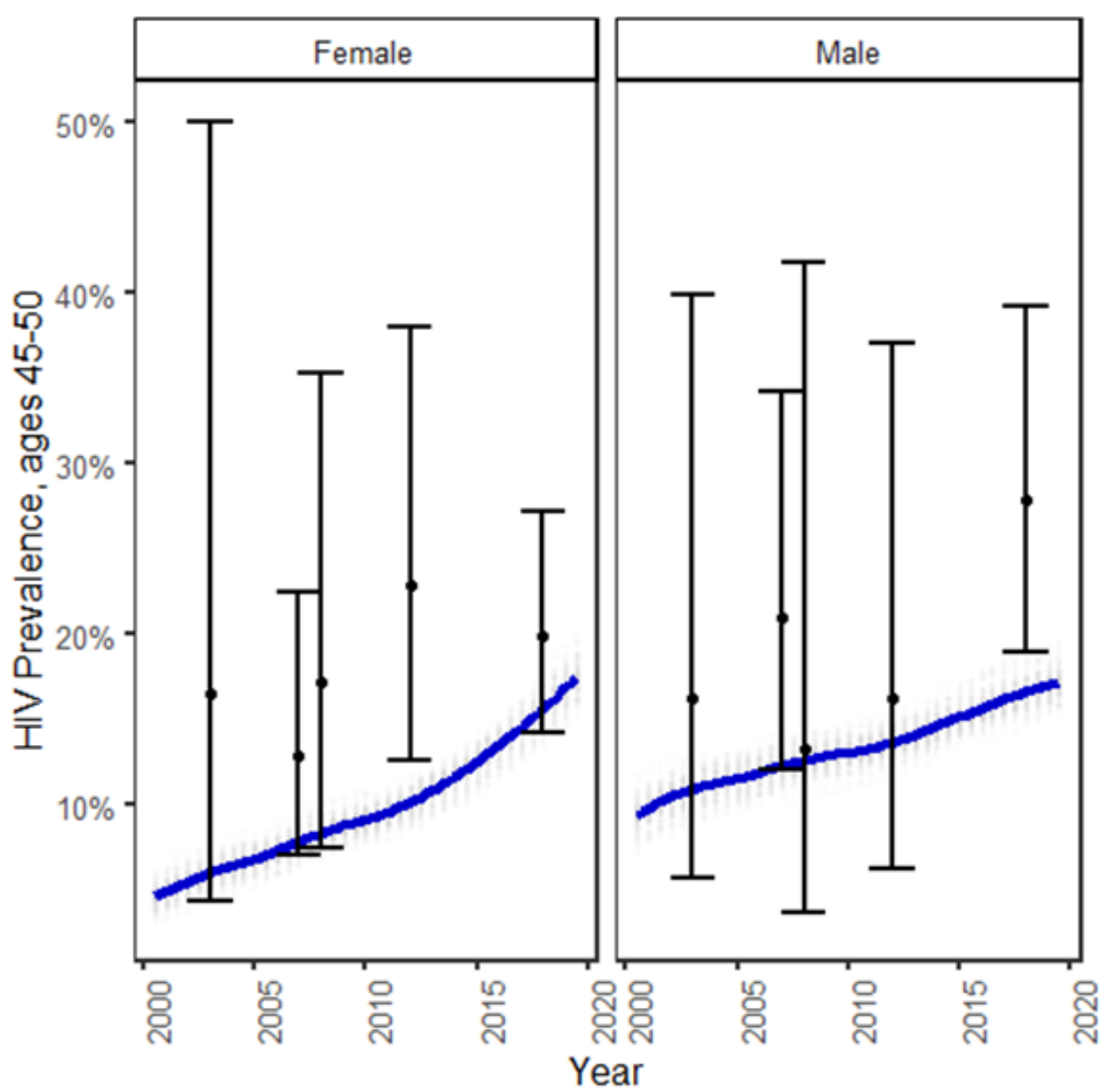

Female
Male
50%
40%
30%
20%
10%
HIV Prevalence, ages 45-50
2000
2005
2010
2015
2020
Year

Table S6. Key model parameters.

Select model parameters used to fit the EMOD-HIV transmission model to survey data on prevalence and ART coverage from Kenya. Median and interquartile ranges (IQRs) reported for all dynamic parameters used in the calibration process from 100 best-fitting parameter sets. †

Parameter	Parameter Description	Fitted median	(IQR)
ARTLinkMax	Maximum probability of linkage to ART	0.999	(0.978, 1.000)
ARTLinkMid	Year of ART linkage (given eligibility), that is, time of the inflection point in the sigmoid trend.	2003.008	(2002.920, 2003.837)
AcuteDurationMonths	The time since infection, in months, over which the Acute_Stage_Infectivity_Multiplier is applied to coital acts occurring in that time period.	1.000	(1.000, 1.331)
CircumcisionReducedAcquire	The reduction of susceptibility to STI by voluntary male medical circumcision (VMMC).	0.600	(0.598, 0.600)
Homa_BayInfrmlCondomsMax	Maximum rate of condom use in informal relationships in Homa Bay	0.231	(0.230, 0.233)
Homa_BayLOWRisk	Proportion of the population that is low-risk in Homa Bay	0.557	(0.549, 0.559)
Homa_BayTrnsCondomsMax	Maximum rate of condom use in transitory relationships in Homa Bay	0.244	(0.232, 0.245)
InfmrlFormRate	Informal relationship formation rate	0.000	(0.000, 0.000)
InfrmlCondomMid	Year midpoint of logistic scale-up of condom use in informal relationships	1993.007	(1991.629, 1998.680)
InfrmlCondomRate	Rate of logistic scale-up of condom use in informal relationships	2.661	(1.953, 2.909)
InfrmlCondomsMax	Maximum rate of condom use in informal relationships	0.216	(0.215, 0.217)
InfrmlDurHet	Heterogeneity in duration of informal relationships	0.750	(0.750, 0.750)
KisiiInfrmlCondomsMax	Maximum rate of condom use in informal relationships in Kisii	0.231	(0.226, 0.232)
KisiiLOWRisk	Proportion of the population that is low-risk in Kisii	0.938	(0.935, 0.939)
KisiiTrnsCondomsMax	Maximum rate of condom use in transitory relationships in Kisii	0.375	(0.369, 0.376)
KisumuInfrmlCondomsMax	Maximum rate of condom use in informal relationships in Kisumu	0.182	(0.181, 0.183)
KisumuLOWRisk	Proportion of the population that is low-risk in Kisumu	0.764	(0.762, 0.765)
KisumuTrnsCondomsMax	Maximum rate of condom use in transitory relationships in Kisumu	0.338	(0.337, 0.347)
LogBaseInfectivity	The probability of transmission when none of the transmission multipliers apply to a particular coital act.	0.002	(0.002, 0.002)

Parameter	Parameter Description	Fitted median	(IQR)
MaleToFemaleOld	Male-to-female relative risk of infection among older individuals	2.012	(1.506, 2.132)
MaleToFemaleYoung	Male-to-female relative risk of infection among young individuals	1.116	(1.000, 1.242)
MaxInfrmlFLOW	Maximum number of informal relationships among low-risk females	1.311	(1.255, 1.315)
MaxInfrmlFMED	Maximum number of informal relationships among medium-risk females	2.050	(2.002, 2.351)
MaxInfrmlMLOW	Maximum number of informal relationships among low-risk males	1.165	(1.159, 1.203)
MaxInfrmlMMED	Maximum number of informal relationships among medium-risk males	2.530	(2.442, 2.735)
MaxMrtlFMED	Maximum number of marital relationship among medium-risk females	1.139	(1.136, 1.159)
MaxMrtlMMED	Maximum number of marital relationship among medium-risk males	1.302	(1.268, 1.316)
MaxTrnsFLOW	Maximum number of transitory relationships among low-risk females	1.599	(1.580, 1.625)
MaxTrnsFMED	Maximum number of transitory relationships among medium-risk females	2.943	(2.891, 3.000)
MaxTrnsMLOW	Maximum number of transitory relationships among low-risk males	1.599	(1.588, 1.677)
MaxTrnsMMED	Maximum number of transitory relationships among medium-risk males	2.557	(2.475, 2.879)
MigoriInfrmlCondoms Max	Maximum rate of condom use in informal relationships in Migori	0.186	(0.185, 0.189)
MigoriLOWRisk	Proportion of the population that is low-risk in Migori	0.795	(0.793, 0.797)
MigoriTrnsCondomsM ax	Maximum rate of condom use in transitory relationships in Migori	0.249	(0.248, 0.257)
MrtlCondomMax	Maximum rate of condom use in marital relationships	0.192	(0.191, 0.193)
MrtlCondomMid	Year midpoint of logistic scale-up of condom use in marital relationships	2003.1 31	(1999.017, 2005.000)
MrtlCondomRate	Rate of logistic scale-up of condom use in marital relationships	2.971	(2.595, 3.000)
MrtlFormRate	Marital relationship formation rate	0.000	(0.000, 0.000)
NyamiraInfrmlCondo msMax	Maximum rate of condom use in informal relationships in Nyamira	0.096	(0.093, 0.097)
NyamiraLOWRisk	Proportion of the population that is low-risk in Nyamira	0.902	(0.900, 0.910)

Parameter	Parameter Description	Fitted median	(IQR)
NyamiraTrnsCondoms Max	Maximum rate of condom use in transitory relationships in Nyamira	0.309	(0.304, 0.313)
PrExInfrmlFemLOW	Probability of potential for extra-relational informal relationship among low-risk females	0.366	(0.364, 0.371)
PrExInfrmlFemMED	Probability of potential for extra-relational informal relationship among medium-risk females	0.401	(0.401, 0.407)
PrExInfrmlMaleLOW	Probability of potential for extra-relational informal relationship among low-risk males	0.244	(0.222, 0.249)
PrExInfrmlMaleMED	Probability of potential for extra-relational informal relationship among medium-risk males	0.382	(0.379, 0.389)
PrExTrnsFemLOW	Probability of potential for extra-relational transitory relationship among low-risk females	0.033	(0.033, 0.033)
PrExTrnsFemMED	Probability of potential for extra-relational transitory relationship among medium-risk females	0.460	(0.450, 0.468)
PrExTrnsMaleLOW	Probability of potential for extra-relational transitory relationship among low-risk males	0.330	(0.328, 0.332)
PrExTrnsMaleMED	Probability of potential for extra-relational transitory relationship among medium-risk males	0.593	(0.589, 0.611)
PreARTLinkMax	Maximum probability of linkage to pre-ART care	0.715	(0.708, 0.737)
PreARTLinkMid	Year midpoint of logistic scale-up of pre-ART linkage	1996.709	(1995.685, 1998.896)
PreARTLinkMin	Minimum probability of linkage to pre-ART care	0.435	(0.416, 0.442)
RiskAssortivity	Risk assortivity	0.663	(0.648, 0.673)
SeedYrHigh	Seed year	1986.562	(1982.000, 1988.000)
SexualDebutAgeFemaleWeibullHeterogeneity	Heterogeneity parameter of Weibull distribution of female age of sexual debut	0.086	(0.083, 0.086)
SexualDebutAgeFemaleWeibullScale	Scale parameter of Weibull distribution of female age of sexual debut	16.013	(15.540, 16.038)
SexualDebutAgeMale WeibullHeterogeneity	Heterogeneity parameter of Weibull distribution of male age of sexual debut	0.040	(0.040, 0.040)
SexualDebutAgeMale WeibullScale	Scale parameter of Weibull distribution of male age of sexual debut	15.708	(15.155, 16.090)
SiayaInfrmlCondoms Max	Maximum rate of condom use in informal relationships in Siaya	0.160	(0.148, 0.161)
SiayaLOWRisk	Proportion of the population that is low-risk in Siaya	0.729	(0.722, 0.731)
SiayaTrnsCondomsMax	Maximum rate of condom use in transitory relationships in Siaya	0.300	(0.293, 0.304)
TrnsCondomMax	Maximum rate of condom use in transitory relationships	0.243	(0.242, 0.253)

Parameter	Parameter Description	Fitted median	(IQR)
TrnsCondomMid	Year midpoint of logistic scale-up of condom use in transitory relationships	1997.960	(1996.962, 1999.273)
TrnsCondomRate	Rate of logistic scale-up of condom use in transitory relationships	0.999	(0.978, 1.000)
TrnsFormRate	Transitory relationship formation rate	2003.008	(2002.920, 2003.837)

† We utilized previously published version of EMOD-HIV and recalibrated with most recent data. The only modifications to the default parameterization outside of PrEP implementation and HIV test sensitivity (described in the main manuscript text) are detailed in Table S6 above. A full description of all parameters and references available is at: https://docs.idmod.org/projects/emod-hiv/en/latest/parameter-overview.html and elsewhere.

Table S7. Disability weights for estimating disability-adjusted life-years averted

**Health State**	**DALY Weight**	**Reference**
HIV-negative	0	Vos *et al*[16]
HIV and not on ART	0.274	
HIV and on ART	0.078	

Instead of calculating total DALYs associated with each scenario using life tables (i.e. conditional life expectancy by age), we calculated DALYs averted the intervention scenario compared to the baseline scenario as the difference in life years and years lived with disability in the intervention compared to the baseline scenario. Years of life lost (YLL) are captured through as the difference in years of life lived between scenarios, while difference in years lived with disability (YLD) are calculated by applying disability weights to time spent in each health state and subtracting the total YLD in the baseline vs intervention scenario over the analytic time horizon.

2.2 Model calibration to population parameters

Population scaling factor

We applied scaling factors of approximately 60 to each run to increase the size of our modeled population and outcomes to reflect the actual population size in western Kenya. To determine the scaling factor for each simulation, we divided the size of the Kenya 2019 census population by the size of the population in 2019 and multiplied this ratio by the modeled population and outcomes. The size of the scaling factor varied slightly depending on model run due to stochastic variability in modeled population size.

Table S8. Population size by gender, county, and age group in 2009[¥]

Age Group	Men						Women					
	Homa Bay	Kisii	Kisumu	Migori	Nyamira	Siaya	Homa Bay	Kisii	Kisumu	Migori	Nyamira	Siaya
0 - < 1	18,335	18,236	17,457	19,265	10,313	15,093	18,354	17,993	16,926	19,309	10,263	14,860
1 - 4	69,799	69,529	63,054	69,921	41,165	56,269	69,250	69,023	63,172	69,519	40,396	55,901
5 - 9	75,926	76,757	67,083	73,872	46,450	60,966	75,973	75,778	67,779	74,333	46,867	60,710
10 - 14	68,689	68,473	62,706	64,300	42,590	58,296	67,159	68,072	63,359	63,249	42,198	56,248
15 - 19	57,430	59,228	55,597	53,075	36,604	49,220	54,119	60,776	56,741	52,238	36,786	47,825
20 - 24	39,573	41,898	47,281	38,690	24,409	32,725	50,309	58,225	57,649	48,004	34,184	41,443
25 - 29	30,437	32,792	40,964	30,727	19,515	25,961	36,016	42,878	40,614	34,670	27,273	30,135
30 - 34	23,259	26,678	30,412	23,344	16,605	20,359	26,342	30,031	27,515	25,630	19,487	22,328
34 - 39	16,013	21,766	21,251	17,024	14,039	14,793	20,010	26,051	20,611	19,313	17,106	17,932
40 - 44	11,914	15,718	15,145	12,170	10,470	11,118	16,513	18,360	16,894	14,773	11,377	16,082
45 - 49	11,124	16,797	13,361	10,549	11,318	10,390	15,248	19,181	15,298	12,888	11,886	15,486
50 - 54	9,705	12,789	11,251	8,565	8,379	9,079	12,942	14,136	12,504	10,314	8,703	14,541
55 - 59	8,159	9,527	8,718	6,399	5,999	8,414	9,833	9,528	9,175	7,692	5,819	12,265
60 - 64	6,989	7,395	7,054	5,250	5,026	7,712	8,587	7,654	7,597	6,000	5,107	11,081

65 - 69	4,325	4,637	4,163	3,382	3,094	5,107	5,957	5,320	5,402	4,508	3,322	7,732
70 - 74	4,029	3,945	3,777	2,907	2,753	5,175	5,355	5,017	4,757	3,524	3,153	7,173
75 - 79	2,835	2,743	2,392	2,033	1,778	3,549	3,891	3,338	3,356	2,968	1,919	5,464
80 - 99	3,726	3,701	2,821	2,624	2,393	4,159	5,316	5,891	4,615	3,636	3,475	6,155

¥Source: Kenya National Bureau of Statistics, 2009 Census

Table S9a. Age-specific population fertility rates in Kenya 1950-2049[¥]

	Age-specific fertility rates (births per 1,000 women)						
Year	15-19	20-24	25-29	30-34	35-39	40-44	45-49
1950-1955	169.1	351.6	338.1	284.3	203.5	110.7	38.9
1955-1960	175.9	365.9	351.9	295.8	211.8	115.2	40.5
1960-1965	182.3	379.1	364.5	306.5	219.4	119.4	41.9
1965-1970	183.3	381.2	366.6	308.2	220.6	120	42.2
1970-1975	180.6	375.5	361.1	303.6	217.3	118.3	41.5
1975-1980	172.7	359.1	345.3	290.3	207.8	113.1	39.7
1980-1985	163.1	339.2	326.2	274.2	196.3	106.8	37.5
1985-1990	147.8	307.3	295.5	248.4	177.8	96.8	34.0
1990-1995	115.3	268.9	252.0	206.8	161.6	73.4	52.0
1995-2000	111.5	260.7	253.3	196.2	143.3	62.4	42.7
2000-2005	104.2	243.6	236.7	183.4	133.9	58.3	39.9
2005-2010	97.1	227.1	221.4	170.6	123.7	53.6	36.5
2010-2015	86.2	201.9	202.3	149.2	102.1	42.4	27.9
2015-2020	75.1	176.5	179.8	129.6	85.8	34.8	22.4
2020-2024	69.9	165.1	171.8	120.2	76.2	29.9	18.6
2025-2029	65.0	154.7	164.8	112.6	68.5	26.0	15.5
2030-2034	60.6	145.7	159.1	106.7	62.5	22.9	13.1
2035-2039	56.2	137.2	153.6	101.8	57.6	20.4	11.0
2040-2044	52.2	129.7	149.3	98.2	53.8	18.5	9.4

[¥]**Source**: 2019 World Population Prospects

Table S9b. Age-specific mortality rates among in Kenya 1950-2049 by gender [¥]

		Age-specific mortality rates (%)						
Sex	Year	15-19	20-24	25-29	30-34	35-39	40-44	45-49
Women	1997.5	0.136	0.191	0.244	0.294	0.361	0.453	0.542
	2002.5	0.116	0.167	0.215	0.259	0.321	0.408	0.493
	2007.5	0.100	0.145	0.188	0.229	0.286	0.367	0.449
	2012.5	0.085	0.127	0.166	0.202	0.254	0.331	0.408
	2017.5	0.073	0.110	0.145	0.178	0.226	0.298	0.371
	2022.5	0.063	0.096	0.128	0.158	0.201	0.268	0.338
	2027.5	0.054	0.084	0.112	0.139	0.179	0.241	0.307
	2032.5	0.046	0.073	0.099	0.123	0.160	0.217	0.280
	2037.5	0.039	0.064	0.087	0.108	0.142	0.196	0.254
	2042.5	0.034	0.055	0.076	0.096	0.126	0.176	0.231
Men	1997.5	0.165	0.253	0.288	0.345	0.433	0.551	0.713
	2002.5	0.142	0.219	0.251	0.304	0.385	0.494	0.648
	2007.5	0.121	0.189	0.219	0.267	0.343	0.443	0.589
	2012.5	0.104	0.163	0.191	0.235	0.305	0.398	0.535
	2017.5	0.089	0.141	0.166	0.207	0.271	0.357	0.486
	2022.5	0.076	0.122	0.145	0.182	0.241	0.320	0.441
	2027.5	0.065	0.106	0.126	0.160	0.215	0.287	0.401
	2032.5	0.056	0.091	0.110	0.141	0.191	0.258	0.364
	2037.5	0.048	0.079	0.096	0.124	0.170	0.231	0.331
	2042.5	0.041	0.068	0.083	0.109	0.151	0.208	0.300

[¥]**Source**: 2019 World Population Prospects

Figure S4. Model predicted population growth by sex

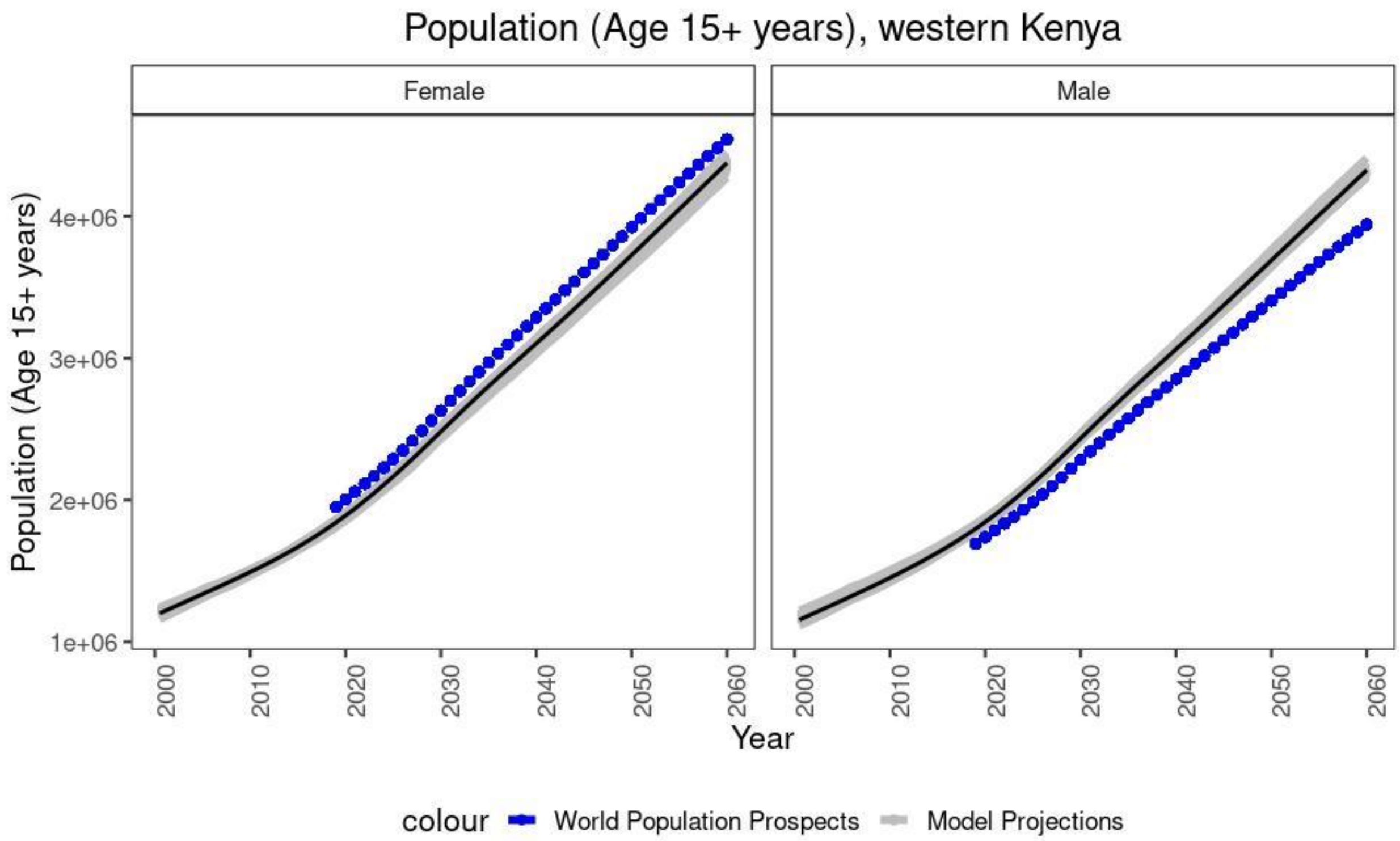

Section 3 | Additional Results

3.1 Sensitivity analyses

Higher MOH costs: We applied a 15% markup to the MOH-perspective costs to account for regulatory oversight, quality assurance, pharmacovigilance, data reporting integration, and referral pathway strengthening which increased the ICER to $339 per DALY averted (vs $212 per DALY averted in the main analysis).
PEPFAR interruption scenario: 20% reduction in testing and ART linkage. We assessed an additional PEPFAR interruption scenario in which the Kenya MOH covers 50% of the funding gaps in HIV testing and treatment, resulting in a 20% reduction in services instead of 41%

Table S10: Sensitivity analyses: higher MOH costs and PEPFAR interruptions

	**Higher MOH costs scenario**	**PEPFAR interruption scenario (20% reduction in testing and ART linkage)**
**PEP coverage (%)**	0.7% (0.7% - 0.8%)	0.7% (0.6% - 0.7%)
**PrEP coverage (%)**	0.3% (0.2% - 0.3%)	0.2% (0.2% - 0.3%)
**HIV infections averted (%)**	13.9% (10.1% -17.1%)	12.8% (10.1% - 15.7%)
**HIV deaths averted (%)**	4.3% (2.0% - 6.9%)	4.0% (2.6% - 5.3%)
**DALYs averted**	36,304 (-39,887 – 108,288)	99,589 (23,760 – 159,595)
**Incremental cost-effectiveness ratio (ICER)**	$339 ($61 - $2,223)	Cost-saving

Broader PEP and PrEP targeting: We conducted structural sensitivity analyses varying uptake and targeting assumptions. In these scenarios, we remove all targeting assumptions and assume all individuals with a non-marital partner are eligible for PrEP and PEP and initiate at equal rates. We maintain the sex ratio observed in our pilot study

Table S11a. Uptake for individuals aged 15-49 with any condomless sex act with a non-marital partner

	PrEP	PEP
Low coverage	1%	2%
Medium coverage	1%	3%
High coverage	1%	4%
Highest coverage	1%	5%

Table S11b. Health and economic impact of broad PEP and PrEP targeting

		Broad targeting			
	Main scenario	Low coverage	Medium coverage	High coverage	Highest coverage
PEP coverage (%)	0.7%<br>(0.7% - 0.8%)	0.6%<br>(0.6% - 0.6%)	0.9%<br>(0.8 % - 0.9%)	1.1%<br>(1.1% - 1.2%)	1.4%<br>(1.3% - 1.5%)
PrEP coverage (%)	0.3%<br>(0.2% - 0.3%)	0.3%<br>(0.3% - 0.3%)	0.3%<br>(0.3% - 0.3%)	0.3%<br>(0.3% - 0.4%)	0.4%<br>(0.3% - 0.4%)
HIV infections averted (%)	13.9%<br>(10.1% -17.1%)	8.8%<br>(4.2% - 12.5%)	11.1%<br>(7.9% - 14.5%)	13.6%<br>(9.7% - 17%)	15.3%<br>(12.0% - 18.4%)
HIV deaths averted (%)	4.3%<br>(2.0% - 6.9%)	3.1%<br>(0.7% - 5.3%)	3.6%<br>(1.4% - 5.7%)	4.5%<br>(2.3% - 6.7%)	5.1%<br>(2.5% - 7.7%)
ICER	$212<br>($15 - $1409)	$440<br>($165 - $6703)	$580<br>($223 - $5548)	$647<br>($262 - $11601)	$812<br>($316 - $6414)

3.2 Replacing daily oral PrEP and PEP with a long acting monthly oral pill

To understand the impact of anticipated novel long acting PrEP/PEP products, we evaluated a scenario where we replaced daily oral PrEP/PEP with MK-8527, a monthly oral pill currently in clinical trials. We set the effectiveness of both PEP and PrEP using MK-8527 at 95%, and assumed the cost of the monthly pill to be $2.88, inclusive of a 15% mark up for supply chain.

The scenario averted 14.1% (10.1% - 17.7%) HIV infections over 10 years and averted 4.6% (1.7% - 7.3%) deaths and 36,257 DALYs over 35 years. Additional details can be found in Section 3.3.

Table S12. Incremental cost-effectiveness ratio using MK-8527 at varying costs

Cost of MK-8527	$2.88	$5.76	$11.52
Incremental cost-effectiveness ratio	Cost-saving	$269/DALY averted<br>(95% UI: $14 – 2356)	$1231/DALY averted<br>(95% UI $324 - $11159)

3.3 Additional Results

Table S13a. HIV infections averted among ages 15-49 in each scenario (2026 - 2036)

Year	Pilot study	PEP only	High efficacy PEP And PrEP	Low efficacy PEP and PrEP	PrEP/PEP provision using MK-8527	Interrupted testing (PEPFAR cuts)	Interrupted testing and ART (PEPFAR cuts)
2026	462	425	435	403	501	401	807
2027	659	589	630	591	713	753	1276
2028	922	832	885	754	910	856	1650
2029	960	741	991	799	916	921	1852
2030	915	741	911	830	862	902	1953
2031	927	769	1001	850	941	897	1977
2032	878	679	845	765	886	969	2201
2033	938	785	974	808	889	903	2320
2034	853	705	878	778	902	903	2335
2035	821	682	821	712	841	768	2353
2036	764	624	752	718	815	778	2366
**TOTAL**	**9099**	**7573**	**9122**	**8008**	**9177**	**9051**	**21089**

Table S13b. HIV deaths averted among ages 15-49 in each scenario (2026 - 2061)

Year	Pilot study	PEP only	High efficacy PEP And PrEP	Low efficacy PEP and PrEP	PrEP/PEP provision using MK-8527	Interrupted testing (PEPFAR cuts)	Interrupted testing and ART (PEPFAR cuts)
2026	-96	-51	-92	-68	-93	-72	-89
2027	-69	-63	-52	-92	-110	-78	-86
2028	-18	-38	-51	-44	-24	-45	18
2029	-27	-6	-8	-18	-7	31	12
2030	74	82	78	87	73	59	111
2031	148	125	131	125	146	138	274
2032	128	98	134	122	152	108	315
2033	167	128	153	178	153	160	209
2034	155	125	171	167	167	158	322
2035	156	128	129	155	189	151	368
2036	157	160	148	159	166	170	475
2037	171	120	149	151	149	133	550
2038	130	113	120	118	142	103	587
2039	123	116	144	113	116	128	616
2040	101	77	109	93	81	91	559

2041	115	81	107	89	85	82	703
2042	73	99	85	108	101	98	695
2043	112	82	88	76	98	74	649
2044	21	26	27	12	39	71	726
2045	67	69	107	87	82	68	618
2046	49	53	47	63	54	58	726
2047	46	45	65	69	54	64	674
2048	32	23	56	65	49	76	607
2049	30	32	16	47	30	54	688
2050	40	31	33	15	28	6	636
2051	47	29	46	44	54	32	635
2052	37	36	42	39	37	6	647
2053	21	24	29	10	19	48	614
2054	36	23	41	15	30	33	534
2055	26	16	29	18	27	26	488
2056	36	20	25	28	50	27	532
2057	17	12	18	19	19	40	468
2058	35	29	29	36	31	17	459
2059	23	21	25	19	19	44	444
2060	23	12	26	15	29	22	399
2061	30	20	39	22	29	24	357
**TOTAL**	**2215**	**1896**	**2243**	**2141**	**2264**	**2207**	**16541**

Table S13c. Distribution of PEP initiations per person year by age, sex, and partnership type

	**Number of time PEP was initiated among PEP users (%)**			
	**1**	**2**	**3**	**4+**
ABYM with multiple concurrent partners	76.8%	19.5%	3.3%	0.4%
ABYM 15-24 with multiple partners in the past 3 months	79.0%	17.7%	3.0%	0.3%
AGYW 15-24 with multiple concurrent partners	81.0%	16.4%	2.3%	0.3%
AGYW 15-24 with multiple partners in the past 3 months	81.7%	15.8%	2.2%	0.3%
Men 25-49 with multiple concurrent partners	69.4%	23.8%	5.9%	0.9%
Men 25-49 with multiple partners in the past 3 months	71.1%	22.5%	5.5%	0.9%
Women 25-49 with multiple concurrent partners	50.6%	26.7%	16.7%	6.0%
Women 25-49 with multiple partners in the past 3 months	54.3%	25.0%	15.3%	5.4%

Table S13d. HIV infections averted by age and sex (2026-2036)

	Mean	CI low	CI high	Mean %	CI low	CI high

ABYM 15-24	766	202	1356	19%	6%	31%
Male 25-49	2859	1926	3760	17%	12%	21%
Male 50+	38	-232	389	2%	-13%	19%
AGYW 15-24	2195	1231	3530	13%	8%	19%
Female 25-49	3814	2610	5047	13%	9%	17%
Female 50+	34	-198	242	2%	-15%	15%

Figure S5. Total 35 year undiscounted costs (in $M) by category for each modeled scenario

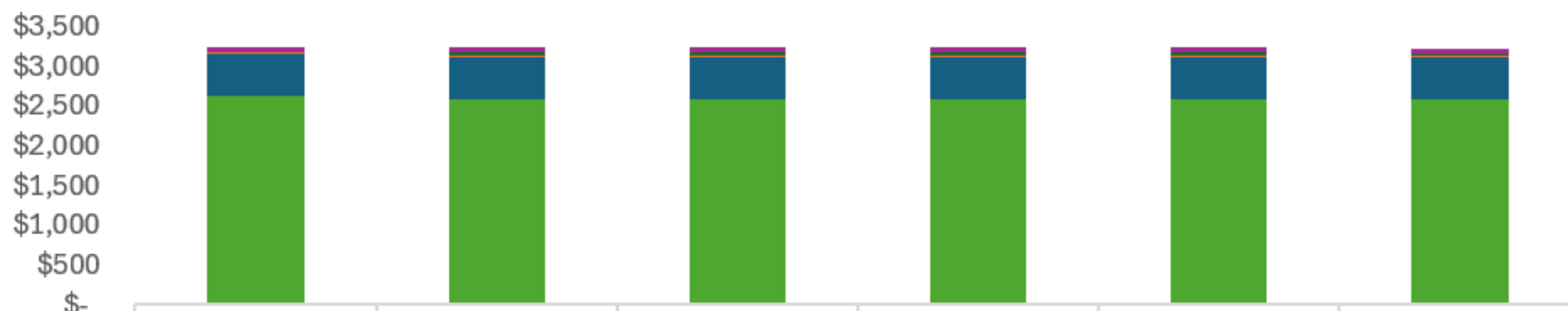

	Baseline	Main scenario	PEP only	High effectivenes of PEP and PrEP	Low effectiveness of PEP and PrEP	PrEP/PEP provision using MK-8527
Total	$3,263	$3,256	$3,259	$3,256	$3,261	$3,233
Background oral PrEP	$86	$86	$86	$86	$86	$86
PrEP	$-	$8	$1	$8	$8	$7
PEP	$-	$33	$34	$33	$33	$14
Illness	$29	$26	$27	$26	$26	$26
Testing	$521	$522	$521	$522	$522	$522
ART	$2,627	$2,580	$2,590	$2,580	$2,585	$2,578

Figure S6. Cost difference by component relative to baseline over 35 years

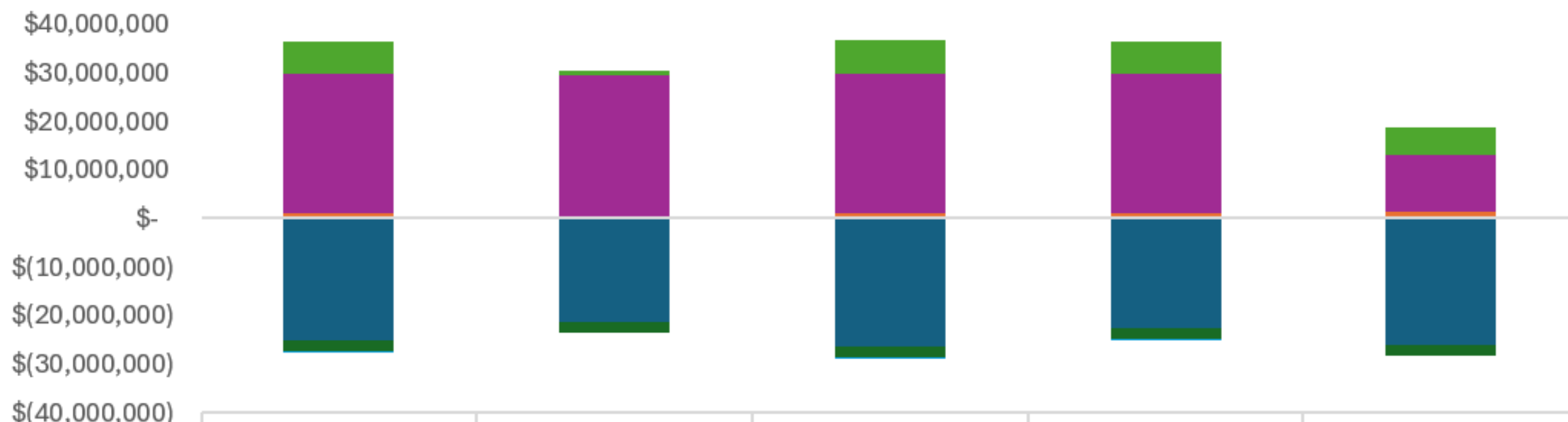

	Main scenario	PEP only	High effectivenes of PEP and PrEP	Low effectiveness of PEP and PrEP	PrEP/PEP provision using MK-8527
Total	$9,372,956	$7,290,115	$8,370,350	$12,127,459	$(9,855,554)
PrEP	$7,171,166	$1,147,221	$7,174,732	$7,164,273	$5,593,797
PEP	$28,570,332	$29,244,774	$28,578,407	$28,557,775	$11,745,092
Background oral PrEP	$(29,816)	$4,769	$(31,967)	$(29,654)	$(40,464)
Illness	$(2,255,331)	$(2,003,332)	$(2,256,436)	$(2,208,437)	$(2,287,991)
Testing	$1,170,093	$343,891	$1,187,966	$1,142,749	$1,300,759
ART	$(25,253,488)	$(21,447,208)	$(26,282,353)	$(22,499,247)	$(26,166,748)

ART   Testing   Illness   Background oral PrEP   PEP   PrEP   Total

Figure S6 presents the cost difference from the baseline scenario for each online PrEP/PEP scenario compared to the baseline counterfactual. Cost savings in ART and HIV-related illnesses are observed across scenarios while costs for PrEP, PEP, and HIV testing increase relative to baseline.

Figure S7. Total PrEP and PEP doses by scenario (2026-2036)

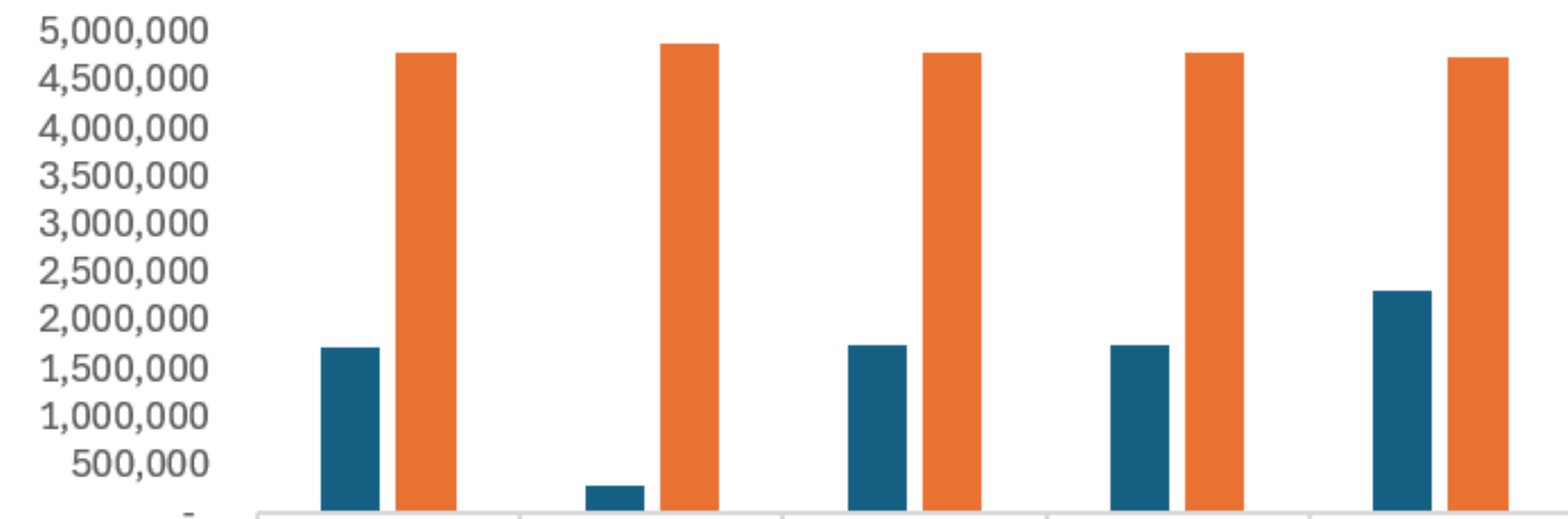

	Main scenario	PEP Only	High effectiveness of PEP and PrEP	Low effectiveness of PEP and PrEP	PEP/PrEP provision using MK-8527
PrEP doses	1,722,259	272,837	1,726,680	1,724,781	2,292,700
PEP doses	4,776,073	4,884,443	4,778,206	4,771,631	4,723,937

PrEP doses   PEP doses

Figure S8. Cost-effectiveness acceptability curve

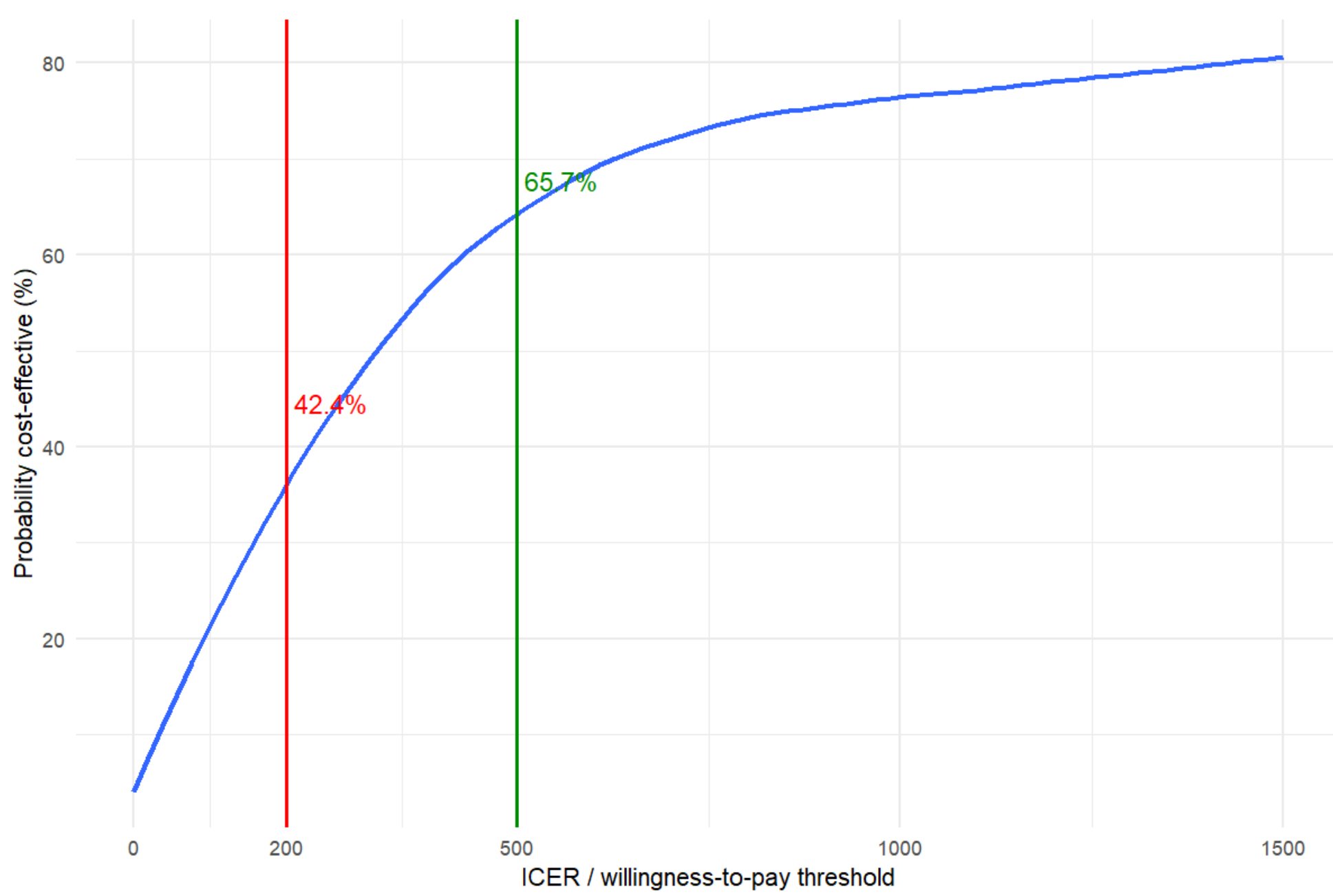

References

1 Taylor D, Durigon M, Davis H, *et al.* Probability of a false-negative HIV antibody test result during the window period: a tool for pre- and post-test counselling. *Int J STD AIDS* 2015; **26**: 215–24.

2 Cox SN, Wu L, Wittenauer R, *et al.* Impact of HIV self-testing for oral pre-exposure prophylaxis scale-up on drug resistance and HIV outcomes in western Kenya: a modelling study. *Lancet HIV* 2024; **11**: e167–75.

3 Kiptinness C, Naik P, Kareithi T. Online delivery of oral HIV pre- and post-exposure prophylaxis: findings from the prospective ePrEP Kenya pilot.

4 Chen Y, Montaño MA, Naik P, *et al.* Incremental Cost of Pre- and Post-exposure Prophylaxis Service Provision via an Online Pharmacy in Kenya. *JAIDS J Acquir Immune Defic Syndr* 2025; **99**: 455–62.

5 Montaño MA, Chen Y, Saldarriaga EM, *et al.* Willingness to pay for HIV pre- and post-exposure prophylaxis services delivered via an online pharmacy in Kenya. *BMC Health Serv Res* 2025; **25**: 576.

6 Turner HC, Lauer JA, Tran BX, Teerawattananon Y, Jit M. Adjusting for Inflation and Currency Changes Within Health Economic Studies. *Value Health* 2019; **22**: 1026–32.

7 Edejer TT-T, World Health Organization, editors. Making choices in health: WHO guide to cost-effectiveness analysis. Geneva: World Health Organization, 2003.

8 Haacker M, Hallett TB, Atun R. On discount rates for economic evaluations in global health. *Health Policy Plan* 2019; : czz127.

9 Eaton JW, Menzies NA, Stover J, *et al.* Health benefits, costs, and cost-effectiveness of earlier eligibility for adult antiretroviral therapy and expanded treatment coverage: a combined analysis of 12 mathematical models. *Lancet Glob Health* 2014; **2**: e23–34.

10 Phillips AN, Cambiano V, Johnson L, *et al.* Potential Impact and Cost-Effectiveness of Condomless-Sex–Concentrated PrEP in KwaZulu-Natal Accounting for Drug Resistance. *J Infect Dis* 2021; **223**: 1345–55.

11 Larson BA, Bii M, Halim N, Rohr JK, Sugut W, Sawe F. Incremental treatment costs for HIV-infected women initiating antiretroviral therapy during pregnancy: A 24-month micro-costing cohort study for a maternal and child health clinic in Kenya. *PLOS ONE* 2018; **13**: e0200199.

12 The Global Fund. The Global Fund. Pooled Procurement Mechanism Reference Pricing: ARVs. https://www.theglobalfund.org/media/3261/core_budgetinginglobalfundgrants_guideline_en.pdf (accessed Sept 8, 2024).

13 Meisner J, Roberts DA, Rodriguez P, *et al.* Optimizing HIV retesting during pregnancy and postpartum in four countries: a cost-effectiveness analysis. *J Int AIDS Soc* 2021; **24**: e25686.

14 Mangale. Comparing the cost of six-month PrEP dispensing with interim HIV self-testing to the standard-of-care three-month PrEP dispensing with clinic-based testing in Kenya.

15 MYDAWA. Email communication: ePrEP Implementation Costs.

16 Vos T, Lim SS, Abbafati C, *et al.* Global burden of 369 diseases and injuries in 204 countries and territories, 1990–2019: a systematic analysis for the Global Burden of Disease Study 2019. *The Lancet* 2020; **396**: 1204–22.
