# Supplementary material for: Cost-effectiveness of providing pre-exposure and post-exposure prophylaxis for the prevention of HIV via online pharmacies in western Kenya: a modelling study
Source: Lancet Glob Health. 2026 Aug 7;14(9):None. doi: 10.1016/j.langlo.2026.103977 (PMC13541651; doi:10.1016/j.langlo.2026.103977)
Supplement: Supplementary file 2 [file mmc2.pdf]

THE LANCET
Global Health

Supplementary appendix 2

This appendix formed part of the original submission and has been peer reviewed.
We post it as supplied by the authors.

Supplement to: Malhotra A, Patel N, Kaftan D, et al. Cost-effectiveness of providing pre-exposure and post-exposure prophylaxis for the prevention of HIV via online pharmacies in western Kenya: a modelling study. *Lancet Glob Health* 2026. https://doi.org/10.1016/j.langlo.2026.103977

Equitable Partnership Declaration

If any questions do not apply to your study, please indicate "N/A" for "not applicable.
For more information on how to complete this form see the Information for Authors document.

Researcher considerations

1. Please detail the involvement that researchers who are based in the country or countries of study had during a) study design; b) clinical study processes, such as processing blood samples, prescribing medication, or patient recruitment; c) data interpretation; and d) manuscript preparation, commenting on all aspects. If they were not involved in any of these aspects, please explain why.

This should include a thorough description of their leadership roles in the study. Are local researchers named in the author list or the acknowledgements, or are they not mentioned at all (and, if not, why)? Please also describe the involvement of early career researchers based in the location of the study. Some of this information might be repeated from the Contributors section in the manuscript. Note: we adhere to ICMJE authorship criteria for naming authors on a paper.

**a) Study design:**<br>CK, TK, and KN contributed to the study conception, provided input on the design of the modelled scenarios to ensure they were aligned with realistic service delivery strategies for Kenya; they also provided input on cost data and payment structure so that scenarios reflected an accurate cost sharing structure between the Kenya Ministry of Health and retail pharmacies.
**b) Clinical study processes:**<br>CK, TK, and KN supported recruitment and implementation operations of the pilot study which was used to inform model inputs.
**c) Data interpretation:**<br>CK, TK, and KN provided input on model results for the discussion section of the manuscript.
**d) Manuscript preparation:**<br>CK, TK, and KN provided feedback on manuscript drafts and approved the final version of the manuscript.

2. How was funding used to remunerate and enhance the skills of researchers in the countries of study? And how was funding used to improve research infrastructure at the study sites?

Potentially effective investments into long-term skills and opportunities within local institutions could include training or mentorship in analytical techniques and manuscript writing, opportunities to lead all or specific aspects of the study, financial remuneration rather than requiring volunteers, and other professional development and educational opportunities.

Improvements to research infrastructure could include funding extended trial designs (eg, platform trials), establishment of long-term contracts for research staff, building research facilities, and setting up local control of funding allocation.

**Skills:** This study was funded by the Bill and Melinda Gates Foundation. In country researchers were provided salary support for their contributions to data collection and management. CK presented the pilot study findings at conferences and was the first author of the main paper reporting empiric findings. In country authors received mentorship in manuscript writing and data analysis and will be authors on several resulting manuscripts from this study.
**Research infrastructure:**<br>N/A

3. How did you safeguard the researchers who implemented the study?

Please describe how you guaranteed safe working conditions for study staff, including provision of appropriate personal protective equipment, protection from violence, and prevention of overworking.

We worked with staff who had implemented the pilot study to obtain empiric data used for this modelling analysis. In the pilot, staff were primarily supervised by our implementing partner, Partners in Health and Research Development. We had regular meetings with staff overseeing primary data collection to ensure they were supported in conducting study activities.

Benefits to the communities and regions of study

4. How does the study address the research and policy priorities of its location?

How were the local priorities determined and then used to inform the research question? Who decided which priorities to take forward? Which elements of the study address those priorities?

Kenya has been a leader in testing and implementing differentiated models of HIV service delivery. The Kenya Ministry of Health (MOH) has identified private pharmacies as a priority service delivery channel for PrEP. In 2023, the MOH established a private sector framework for HIV service delivery. This study provides data on efficiency and health impacts of online PrEP and PEP delivery, which can inform decision making regarding MOH investments in online pharmacy platforms for expansion of coverage of biomedical HIV prevention.

5. How will research products be shared in the community of study?

For instance, will you be providing written or oral layperson summaries for non-academic information sharing? Will study data be made available to institutions in the region(s) of study?

The Lancet Group *encourages authors to translate the summary (abstract) into relevant languages after paper editing; do you intend to translate your summary?*

Study results will be shared with local stakeholders through presentations and written reports. We will also share findings with MYDAWA, Kenya's online pharmacy retailer, who would be the target partner in implementing this strategy. As most meetings and presentations in Kenya are conducted in English, we will not be translating our abstract.

6. How were individuals, communities, and environments protected from harm?

a) How did you ensure that sensitive patient data were handled safely and respectfully? Was there any potential for stigma or discrimination against participants arising from any of the procedures or outcomes of the study?

N/A

b) Might any of the tests be experienced as invasive or culturally insensitive?

N/A

c) How did you determine that work was sensitive to traditions, restrictions, and considerations of all cultural and religious groups in the study population?

N/A

d) Were biowaste and radioactive waste disposed of in accordance with local laws?

N/A

e) Were any structures built that would have impacted members of the community or the environment (such as handwashing facilities in a public space)? If so, how did you ensure that you had appropriate community buy-in?

N/A

f) How might the study have impacted existing health-care resources (such as staff workloads, use of equipment that is typically employed elsewhere, or reallocation of public funds)?

N/A

7. Confirm that local ethics review was sought, and please provide the approval number. If not sought, please explain why.

Local ethical review was obtained for the pilot study (published elsewhere). The present modelling analysis did not engage with human subjects so no approval was sought.

Secondary analyses

8. Have the data analysed in your study been extracted from another source, such as a national survey, rather than being directly collected by the authors of this paper?

Yes, several of our model inputs were obtained from national surveys and published papers.

If the authors of this paper were not involved in data collection, how were the findings interpreted with sufficient contextual knowledge?
The Lancet Group *believe contextual understanding is crucial for informed data analysis and interpretation*.

We take into consideration the contexts in which data were collected, including the biases that may be present. For example, household surveys often underestimate HIV prevalence among men who are more likely to refuse participation, particularly if they are known to have HIV. Additionally estimates of viral suppression are often obtain only among those who attend clinics, thereby not capturing viremia in those lost to follow up. We utilized not only the point estimates but the confidence intervals and calibrate the model simultaneously to several epidemiologic parameters.

---

9. Please provide the title (eg, Dr/Prof, Mr/Mrs/Ms/Mx), name, and email address of an author who can be contacted about this statement.

Name: Dr. Monisha Sharma
Email: msharma1@uw.edu

10. Finally, please provide the title and name of an author from one country of study who has seen and approved this form.

Name: Dr. Kenneth Ngure
